# The Impact of *Akkermansia muciniphila* on Mouse Models of Depression, Anxiety, and Stress: A Systematic Review and Meta-Analysis

**DOI:** 10.2174/011570159X360149250225041829

**Published:** 2025-03-18

**Authors:** Leila Khalili, Gwoncheol Park, Ravinder Nagpal, Pradeep Bhide, Gloria Salazar

**Affiliations:** 1 Department of Health, Nutrition, and Food Sciences, Florida State University, Tallahassee, FL, 32306, USA;; 2 Department of Biomedical Sciences, Institute for Pediatric Rare Diseases, College of Medicine, Florida State University, Tallahassee, FL, 32306, USA

**Keywords:** *Akkermansia muciniphila*, mental health, mouse, depression, anxiety, stress, meta-analysis

## Abstract

**Background:**

*Akkermansia muciniphila* (*A. muciniphila*), a bacterial species within the human gut microbiome, has shown beneficial effects on host health. Emerging research suggests that *A. muciniphila* also influences neurobehavioral domains through the microbiota-gut-brain axis. This meta-analysis evaluates *A. muciniphila*’s impact on depression, anxiety, and stress in mouse models.

**Methods:**

We conducted a systematic search of PubMed, Science Direct, Embase, and Web of Science databases up to March 2024, identifying 15 eligible studies.

**Results:**

Supplementation with *A. muciniphila*, its outer membrane protein (Amuc_1100), and extracellular vesicles (EVs) alleviated anxiety, depressive-like behaviors, and enhanced memory in mice. Compared to controls, intervention groups exhibited reduced anxiety-like behaviors, including increased travel distance in the open-field test (OFT) and more time spent in the lightbox during the light-dark box (LDB) test and open arms in the elevated plus maze (EPM). Depression-like symptoms were reduced, with lower immobility time in the tail suspension and forced swim tests. Memory function also improved, and learning time was reduced in the Y-maze and Barnes circular maze tests. Serotonin levels increased significantly in the serum and hippocampus, while corticosterone levels decreased, though not significantly. The intervention reduced hippocampal and serum inflammatory markers (TNFα, IL1β, IL6) and altered gut microbiome composition, increasing *Akkermansia*, *Roseburia*, *Caldicoprobacter*, and *Lachnospiraceae*.

**Conclusion:**

This meta-analysis provides evidence supporting the health-promoting effects of *A. muciniphila*, one of the next-generation probiotics, in alleviating neuropsychiatric disorders. Given the high prevalence and clinical significance of depression, anxiety, and stress, further investigation into the therapeutic utility of *A. muciniphila* is warranted.

## 1 INTRODUCTION

The gut microbiota, a diverse collection of commensal bacteria, parasites, fungi, archaea, and viruses, is essential for maintaining host health [1]. These microorganisms are integral to human physiology, metabolism, growth, immunity, and development [2] and have an active rather than a passive role in human health and disease [3]. A healthy microbiota (eubiosis) promotes host health by diverse mechanisms, including generating protective gut-derived metabolites (*e.g*., short-chain fatty acids (SCFAs)), maintaining the integrity of the gut barrier (*e.g*., increasing tight junction proteins), and reducing intestinal and systemic inflammation. In contrast, an unhealthy microbiota (dysbiosis) disrupts these protective mechanisms, promoting various diseases, including cardiometabolic diseases (*e.g*., obesity, diabetes, hypertension, and atherosclerosis) [4]. This functional link between the gut microbiota and human health has transformed our understanding of disease development and has paved the way for new diagnostic and therapeutic strategies.

*A. muciniphila*, a commensal bacterium residing in the mucus layer of the intestinal epithelium, has attracted considerable attention in recent years because of its positive effects on energy metabolism, glucose tolerance, and immune system function [5]. *A. muciniphila* has shown promise in managing various metabolic conditions, including obesity, hypertension, diabetes, colitis, and age-related issues. Its capacity to restore the gut microbiota and preserve a healthy gut mucosal barrier underscores its significance in regulating immunity and mitigating inflammation [6-12]. *A. muciniphila* is typically found in healthy individuals and is upregulated by dietary patterns like the Mediterranean diet [13]. However, its levels can be reduced by age, unhealthy diets, and disease conditions [14]. Our recent meta-analysis on the effects of *A. muciniphila* supplementation in animal models of metabolic disorders indicates that both live and heat-killed forms of *A. muciniphila*, as well as its EVs and proteins, can improve lipid profiles, glucose metabolism, liver enzyme levels, gut and systemic inflammation, and body weight [15]. Thus, *A. muciniphila* is a potential probiotic for the management of inflammatory and metabolic disorders. Recent studies have also suggested an association between *A. muciniphila* and the management of neurological and psychiatric disorders symptoms, suggesting the involvement of *A. muciniphila* in the gut-brain axis communication *via* gut-derived metabolites [2].

Mental health disorders have become a considerable global health concern, affecting nearly 970 million people worldwide [16]. Conditions such as depression, anxiety, post-traumatic stress disorder (PTSD), eating disorders, and bipolar disorders can severely compromise individuals' daily functioning and quality of life and, in some cases, lead to suicide. These disorders also cause a significant economic burden to families and healthcare systems, as well as human life losses [17]. The COVID-19 pandemic has further exacerbated these challenges, with quarantine measures, economic downturns, and increased unemployment contributing to a surge in mental health problems. It's anticipated that the pandemic's lasting impact on mental health, particularly in terms of anxiety, depression, and PTSD, will persist in the long term [18].

The role of the microbiota in improving mental health outcomes and the possible use of probiotics to treat the symptoms of these diseases have sparked great interest in recent years. Research in preclinical murine models has uncovered several mechanisms by which the gut microbiota can influence brain function and mental health, including the regulation of the vagus nerve tone, neuro-immune signaling, tryptophan metabolism, neuroendocrine function, and the synthesis of neuroactive compounds [19, 20]. Furthermore, the microbiota influences the activity of neurotransmitters such as serotonin, dopamine, and glutamate, which are essential for brain function [21]. Specific gut bacteria, such as *Roseburia inulinivorans*, *Bacteroides uniformis*, *Faecalibacterium prausnitzii*, and *Eubacterium rectale*, stand out for their positive effects on mental health by producing SCFAs and modulating metabolic pathways [22]. Additionally, gut dysbiosis may contribute to the development and progression of mental disorders [21, 23].

Conventional treatments for mental disorders, such as pharmacotherapy, psychotherapy, and behavioral therapy, often encounter challenges like side effects and patient non-compliance [24, 25]. As a result, there is growing interest in alternative approaches such as acupuncture, meditation, and natural remedies [24]. Natural dietary products, including probiotics, have emerged as promising interventions for managing neuropsychiatric disorders by virtue of their potential to influence the gut microbiota [26]. Moreover, diets rich in vegetables, fruits, and fiber have been linked to improved mental health outcomes, while tryptophan-rich diets have shown associations with reduced depression and enhanced cognition [19, 27, 28].

This meta-analysis aims to provide an overview of the role of *A. muciniphila* in managing neurobehavioral disorders in mouse models of depression, anxiety, and stress. The findings revealed significant beneficial effects of *A. muciniphila* in neurobehavioral disorders.

## 2 MATERIALS AND METHODS

The present research was performed according to PRISMA (Preferred Reporting Items for Systematic Reviews and Meta-Analyses) guidelines and was registered in advance in PROSPERO (CRD42024550582).

### 2.1 Search Strategy

Relevant studies were identified through a search of publications available for retrieval until March 2024 in databases, including PubMed, Science Direct, Embase, and Web of Science, as we previously reported [15]. The search’s objective was to find studies examining *A. muciniphila*’s effects on mental health in mouse models of psychiatric disorders. The search was conducted using the following terms: (*Akkermansia* OR *Akkermansia muciniphila* OR *A. muciniphila* OR *Akk*) AND (Mental disorder OR Depression OR Anxiety OR Stress) AND (Mouse OR Mice). Only studies published in English were included in the review.

### 2.2 Inclusion and Exclusion Criteria

Studies were included if 1) they were conducted in mice, 2) they assessed mental disorders, and 3) they supplemented animals with any form of *A. muciniphila* (live, heat-killed, EVs or protein extracts). Conversely, studies were excluded if they provided insufficient information on the experimental design and mental health outcomes. Other excluded studies were narrative reviews, meta-analyses, and articles lacking original research.

### 2.3 Characteristics of Included Studies

**Fig. (1):**
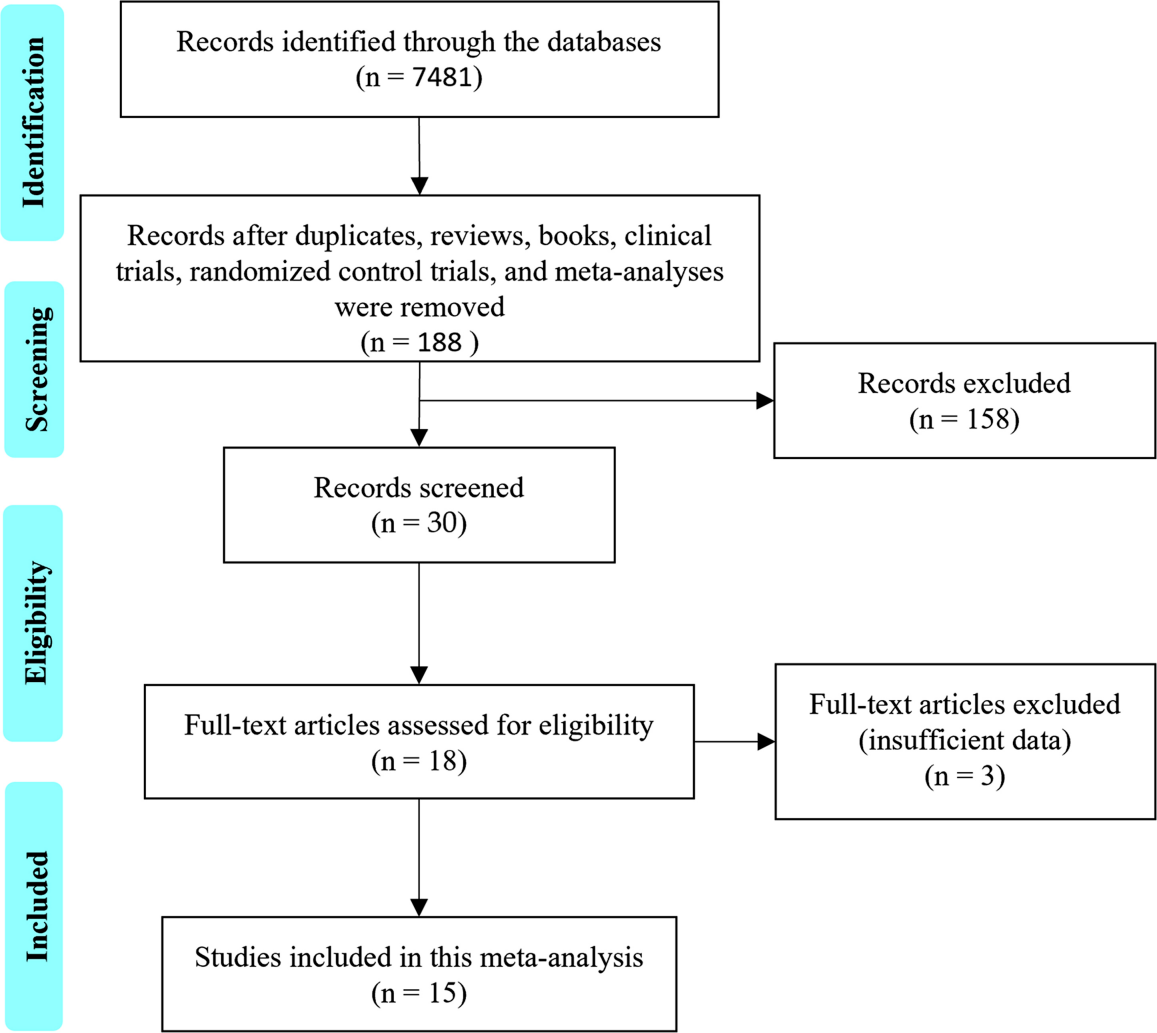
The PRISMA flow diagram shows the search strategy used in this study. Studies of interest were located through extensive searches in databases such as Science Direct, Embase, PubMed, and Web of Science until March 2024. Studies investigating *A. muciniphila’s* effect on mental health in mouse models of depression, anxiety, and stress were considered for inclusion.

The initial search yielded 7,481 articles (ranging from 2000 to 2024). After assessing the articles based on the inclusion and exclusion criteria and removing reviews, books, clinical trials, randomized controlled trials, and meta-analyses, 30 articles were considered for inclusion. Of these, only 18 articles provided original research investigating the effects of *A. muciniphila* on mental health in mouse models of mental disorders. Upon further review of titles and abstracts, 3 studies with insufficient data were excluded, leaving 15 studies presenting sufficient information for data extraction (Fig. **1**).

Publication bias was assessed using the Egger and Begg tests, showing little to no bias, except for the time in the lightbox (Table **S1**). The observed bias could result from the limited studies measuring this outcome (two studies).

Table **S2** shows the studies’ characteristics, including the mouse sex and age, the *A. muciniphila* strain and treatment conditions, and outcomes. Fourteen studies used C57BL/6 mice, and one used the APP/PS1 (Alzheimer's disease model). Regarding gender, most of the studies used males (12 studies), two were conducted in females [29, 30], and only one used both sexes [31]. The age of the animals at the beginning of the study ranged between 5 and 12 weeks. One study reported the weight but not the age of the mice [32], and one used juvenile mice (P21) [33].

Regarding mental health outcomes, anxiety and depression phenotypes were induced in a variety of experimental models, including depression induced by alcohol and lipopolysaccharide (LPS) treatment (mALSP) [29], antibiotic-induced anxiety and depression [34], chronic restraint stress (CRS) depression [32, 33], healthy mice [34, 35], chronic unpredictable mild stress (CUMS) [36, 37], learning and memory impairment in high-fat diet (HFD)-induced obesity [38], traumatic brain injury (TBI) [39], National Institute on Alcohol Abuse and Alcoholism (NIAAA) model [32], chronic alcohol *via* gavage [32], depressive-like behavior induced by CUMS [32], sleep-deprived [40], HFD-induced metabolic disorders [30], neonatal maternal separation (NMS) inflammatory bowel disease (IBD) [31], *Citrobacter rodentium* infection, post-infectious-IBD model [31], Alzheimer’s disease [41], and antibiotic-treated [42] models.

Regarding treatment with *A. muciniphila*, eight of the included studies utilized *A. muciniphila* ATCC^®^ BAA-835™ strain, one used the DMS 22959 strain [32], one used a Chinese strain [41], and five did not report the strain of *A. muciniphila* used [29, 32, 34, 36, 39]. Only one study used pasteurized *A. muciniphila*; three studies used the membrane protein Amuc_1100; two studies used EVs and the rest of the included studies used live *A. muciniphila*.

Behavioral outcomes were measured for anxiety, depression, and learning. Anxiety was measured using the following tests: the light-dark box (LDB), open field test (OFT), and the elevated plus maze (EPM) test. For the OFT, most of the studies measured the total distance traveled. Some studies also measured the number of entries into the center, the time spent in the center, and the time spent in the periphery of the box. For the LDB test, outcomes were measured for time spent in the light chamber and the number of entries into the lightbox. For the EPM test, outcomes were measured for time spent in the open arms and the frequency of entry into the open arms. One study also used the hole board test to assess anxiety and stress responses to an unfamiliar environment [31]. Depression-like behavior was assessed based on the immobility time in assays of learned helplessness, such as the tail suspension test (TST) and the forced swim test (FST). Two studies also measured hedonic behaviors using a sucrose preference test [30, 43].

Memory outcomes were measured using the Y-maze for working memory [30, 31], novel object recognition (NOR) [31, 40], contextual fear-conditioning test, and Barnes circular maze test [38]. The novel location recognition (NLR) test was used in only one study [31]. Moreover learning was measured by Y-maze [41] and Barnes circular maze [38] tests.

### 2.4 Statistical Analyses

Data analysis was performed as previously reported [15] using STATA18 (StataCorp, College Station, TX, USA) by following PRISMA guidelines recommended for systematic reviews and meta-analyses [44]. We used a restricted maximum likelihood method with a random-effects model [45] for the meta-analysis. The random-effects model was chosen to account for the possibility of missing, unidentified, or unregistered studies. Heterogeneity was evaluated using Cochran's I-squared, Q tests, and Tau-squared with substantial heterogeneity defined as an I-squared value greater than 75% [46]. The combined effect size of each study was reported using the standardized mean difference (SMD) and its 95% confidence interval. (CI). Furthermore, funnel plots, as well as Egger's and Begg's tests, were used to assess publication bias [47, 48]. Figs. (**1**-**8**) were generated using STATA18.

## 3 RESULTS

### 3.1 *A. muciniphila* Alleviates Anxiety-like Behavior

**Fig. (2):**
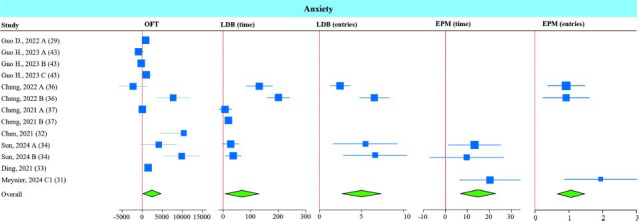
Effect of *A. muciniphila* on anxiety. Forest plot of individual SMD of anxiety tests including total travel distance in the OFT, time spent in light box and number of entries into the lightbox in the LDB test, and time and entries in the open arm in EPM tests of mice receiving *A. muciniphila*. The green diamond represents *p <* 0.05.

As we previously reported [15], the data are presented using the common SMD and 95% confidence interval (95% CI) for the behavioral tests, based on a random-effects model, as shown in Table **S3**. The data show that *A. muciniphila* supplementation significantly increases the total travel distance in the OFT, time spent and entries into the lightbox in the LDB test, and time spent and entries in the open arm in the EPM test (Fig. **2**). Other assessments for the OFT measured in some studies also showed significant alleviation of anxiety-like behavior. For example, there was a reduction in the time in which mice were at the periphery [29] and an increase in the entries and time spent at the center of the field in the OFT [34, 41].

Sub-group analysis of anxiety tests revealed that the effect of Amuc-1100 on improving OFT outcome was stronger than that of live bacteria (Fig. **S1**). Moreover, interventions with both live and non-live interventions (Amuc_1100 and EVs) significantly improved lightbox entries in the LDB test (Fig. **S2A**), though only non-live interventions were significantly effective in increasing the time spent in the lightbox (Fig. **S2B**). However, it should be noted that only one study measured time spent in the lightbox. Sub-group analysis for time spent in the open arms of the EPM showed significant effectiveness of both live and non-live interventions (Fig. **S3**). All studies measuring entries into the open arms (EPM) used non-live interventions (Amuc_1100 and pasteurized bacteria). More studies are needed to conclude that live bacteria are equally effective for these measures.

### 3.2 *A. muciniphila* Improves Depression-like Behavior

**Fig. (3):**
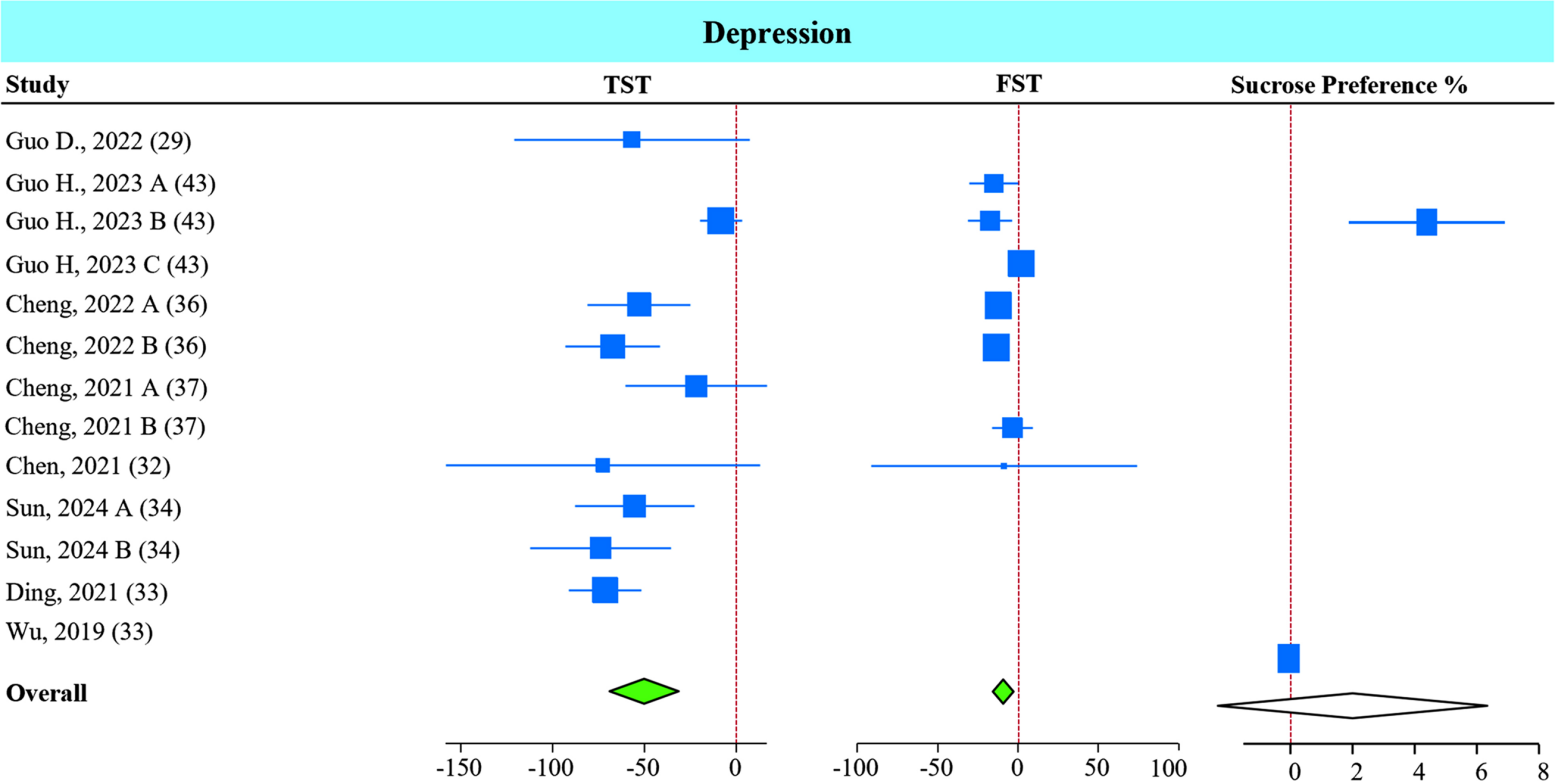
Effect of *A. muciniphila* on depression symptoms. Forest plot of individual SMD of depression tests including tail-suspension test (TST), forced swim test (FST), and sucrose preference test in mice receiving *A. muciniphila*. The green diamond represents *p <* 0.05, and the white diamond represents non-statistical significance.

The immobility time observed in the TST and FST was reduced significantly by *A. muciniphila* supplementation (Fig. **3**). The sucrose preference test was used in only two studies [30, 43], showing non-significant improvement in sucrose intake in the *A. muciniphila* groups.

Sub-group analysis showed that all interventions (live and non-live) improved TST immobility time (Fig. **S4A**), but only the non-live interventions significantly improved immobility time in the FST (Fig. **S4B**).

### 3.3 *A. muciniphila* Improves Cognition Function and Memory

**Fig. (4):**
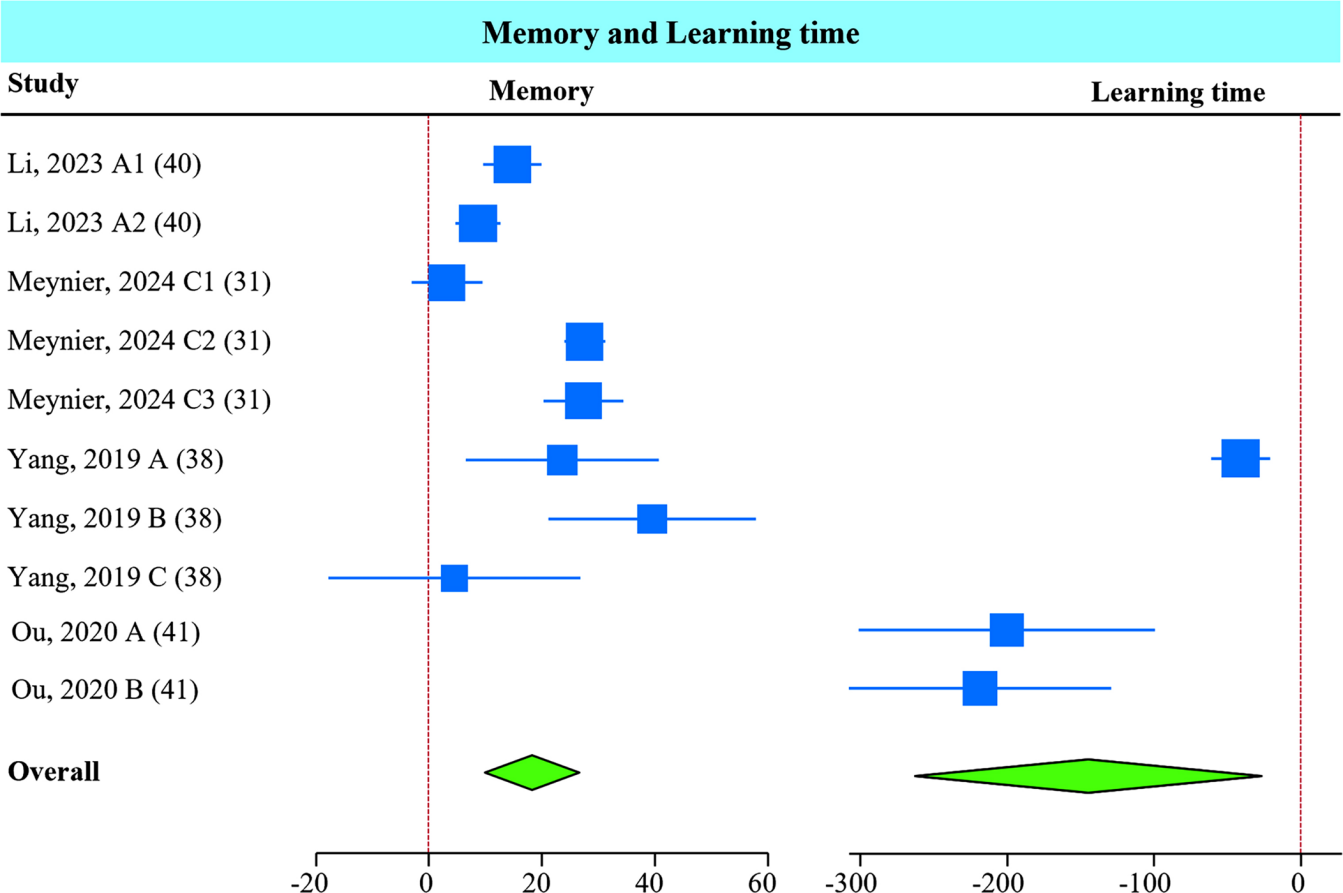
*A. muciniphila* improved memory and learning outcomes. The forest plot shows the standardized mean difference (SMD) for memory, measured by the NOR and Y-maze tests, and learning, assessed using the Y-maze and Barnes circular maze tests, in mice receiving *A. muciniphila*. The green diamond indicates *p* < 0.05.

Memory was evaluated by combining the measurements for the time spent exploring a novel object in the NOR test, spontaneous alternations in the Y-maze test, and percent of freezing time in the contextual fear-conditioning test. Learning was assessed using the Y-maze and Barnes circular maze tests by measuring the time spent learning the task (Fig. **4**). *A. muciniphila* supplementation significantly improved memory and learning outcomes of the combined tests.

Sub-group analysis of memory tests showed that all interventions (live and non-live) improved memory in mice (Fig. **S5**). All studies measuring learning outcomes used live bacteria; thus, no sub-group analysis was performed for this outcome.

### 3.4 *A. muciniphila* Improves Serotonin Levels in the Hippocampus, Serum, and Gut

**Fig. (5):**
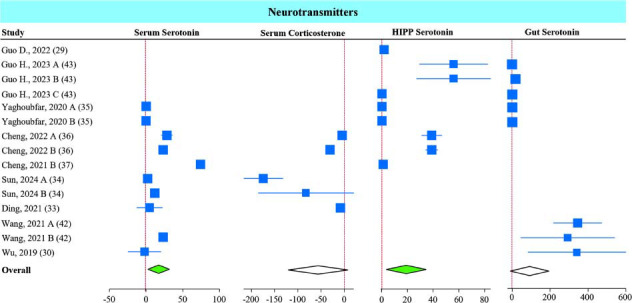
*A. muciniphila* regulates serotonin and corticosterone levels. Forest plot of individual SMD of serotonin levels in serum, HIPP and gut, and serum corticosterone in mice receiving *A. muciniphila*. The green diamond represents *p <* 0.05, and the white diamond non-statistical difference.

Serotonin (5-hydroxytryptamine; 5-HT) is a neurotransmitter implicated in regulating mood, anxiety, memory, as well as gastrointestinal function [49]. Serotonin levels in the serum, hippocampus (HIPP), and gut were evaluated in mouse models of stress, anxiety, and depression-like behavior following *A. muciniphila* administration. Serotonin levels were elevated significantly in the serum and the HIPP, showing an upward trend (*P* = 0.08) in the gut of *A. muciniphila*-treated mice (Fig. **5**). Similarly, the serum level of corticosterone, a stress hormone, was reduced, but the reduction did not reach statistical significance (*P* = 0.08).

Sub-group analysis showed that all interventions (live and non-live) improved serum and HIPP serotonin levels (Figs. **S6A** and **C**). Neither live nor non-live interventions were effective in improving serum corticosterone and gut serotonin levels (Figs. **S6B** and **D**).

### 3.5 *A. muciniphila* Influences Signaling Molecules Associated with Neurotransmitter Function in the Hippocampus, Serum, and Gut

*A. muciniphila’s* effects on factors regulating neuronal plasticity, like brain-derived neurotrophic factor (BDNF) [50] were analyzed in several of the included studies. BDNF regulates serotonin levels and synaptic transmission by regulating serotonin transporter (SERT) expression [51]. Corticosterone, the major glucocorticoid expressed in mice, binds to glucocorticoid receptors (GR) to induce a stress response in the brain. Inflammatory responses induced by stress conditions negatively affect tryptophan metabolism, which is involved in serotonin synthesis. In physiologic conditions, serotonin is synthesized from tryptophan *via* tryptophan hydroxylase 1 (TPH1). Inflammation increases tryptophan 2,3-dioxygenase (IDO), an enzyme that diverts tryptophan into kynurenine synthesis, reducing serotonin expression and causing depression-like symptoms. Furthermore, the ionized calcium-binding adaptor molecule 1 (Iba1) was used as a marker of microglia activation, and the diamine oxidase (DAO), an enzyme expressed in the intestinal epithelium, was used as a marker of intestinal permeability.

**Fig. (6):**
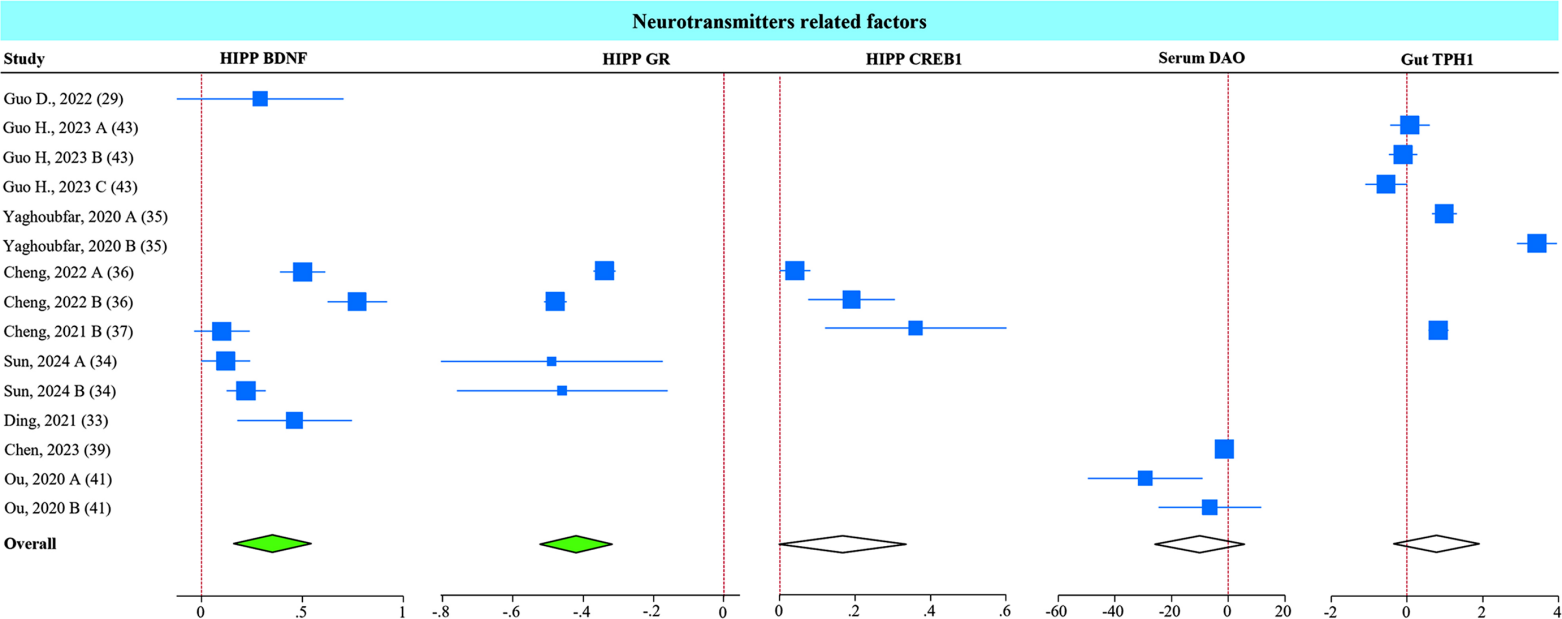
Effect of *A. muciniphila* on neurotransmitter-related factors in the hippocampus, serum, and gut. Forest plot of individual SMD of HIPP BDNF, GR, CREB1, and serum DAO, and gut TPH1 in mice receiving *A. muciniphila*. The green diamond represents *p <* 0.05, and the white diamond non-statistical difference.

Although not all the included studies measured the expression of all these markers, *A. muciniphila* supplementation showed improvement in several of them, including upregulation of BDNF and downregulation of GR in the HIPP (Fig. **6**). cAMP-responsive element-binding protein1 (CREB1), another regulator of neuronal plasticity, showed an upward trend close to significance (*P* = 0.05) in the HIPP. DAO was reduced in the serum, and TPH1 was upregulated in the gut by *A. muciniphila*, trends that did not reach statistically significant.

Sub-group analysis indicated that all interventions (live and non-live) improved BDNF and GR levels in the HIPP (Figs. **S7A** and **B**); however, only non-live interventions significantly improved gut TPH1 levels (Fig. **S7C**). All the studies measuring hippocampus CREB1 used Amuc-1100, and all the studies measuring serum DAO used live bacteria.

### 3.6 *A. muciniphila* Alleviates Inflammation in the Hippocampus, Serum, and Gut

**Fig. (7):**
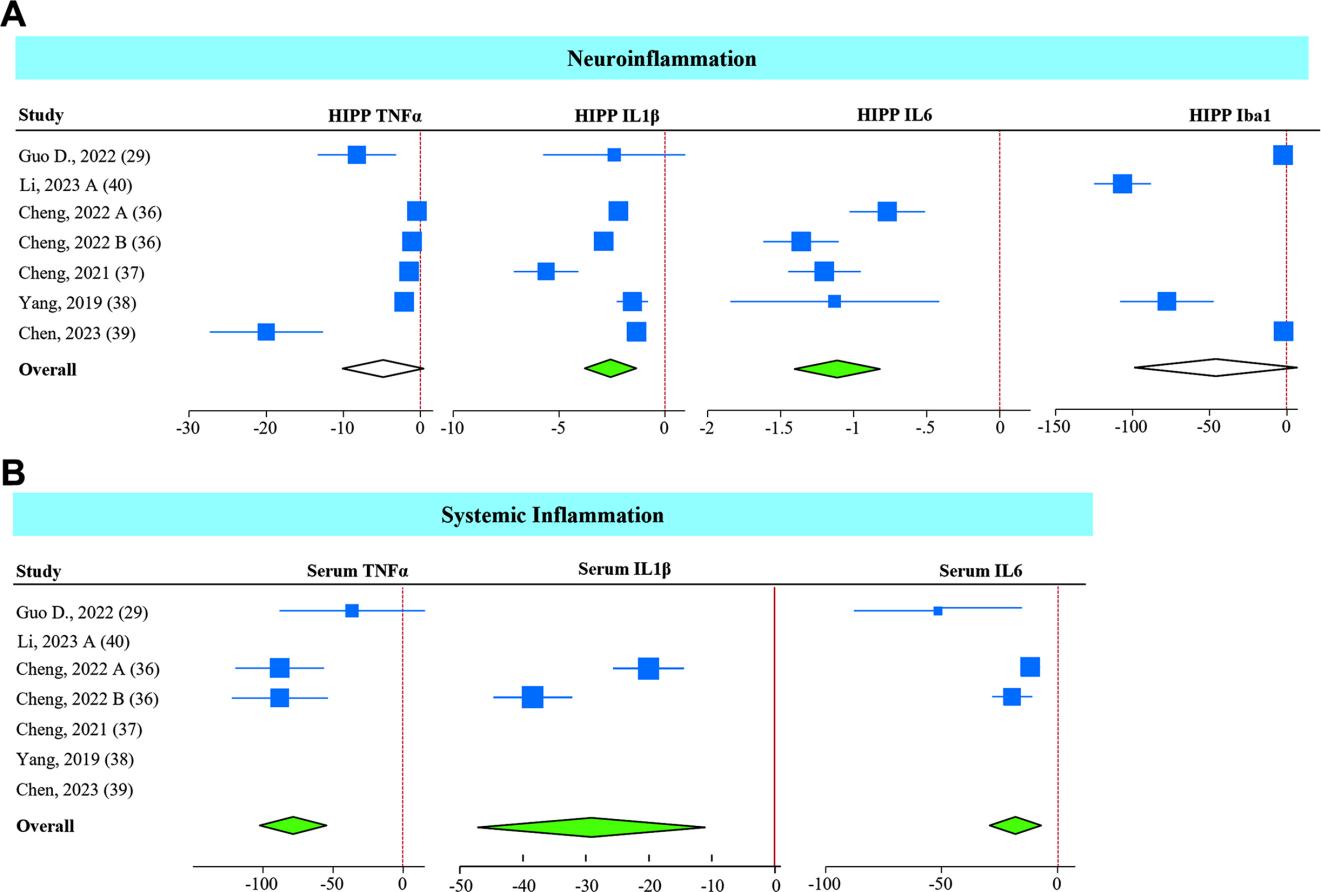
Effect of *A. muciniphila* on inflammation. Forest plot of individual SMD of the hippocampus (**A**) and serum (**B**) inflammatory markers in mice receiving *A. muciniphila*. The green diamond represents *p <* 0.05, and the white diamond non-statistical difference.

Consistent with improvements in serotonin and its regulatory network, *A. muciniphila* supplementation significantly decreased HIPP (Fig. **7A**) and serum (Fig. **7B**) levels of inflammatory markers, including TNFα, IL1β, IL6, and Iba1. However, the reductions in TNFα and Iba1 levels were insignificant (*P* = 0.07 and *P* = 0.08, respectively). The level of serum IL1β was measured only in two studies that showed a significant reduction in its level.

Sub-group analysis showed that all *A. muciniphila* interventions (live and non-live) reduced IL1β and IL6 levels in the HIPP; however, only non-live interventions could significantly reduce HIPP TNFα levels (Figs. **S8A-C**). There were not enough studies to perform a sub-group analysis for the inflammatory markers in the serum.

### 3.7 *A. muciniphila* Influences Tight-junction Protein Expression in the Gut

**Fig. (8):**
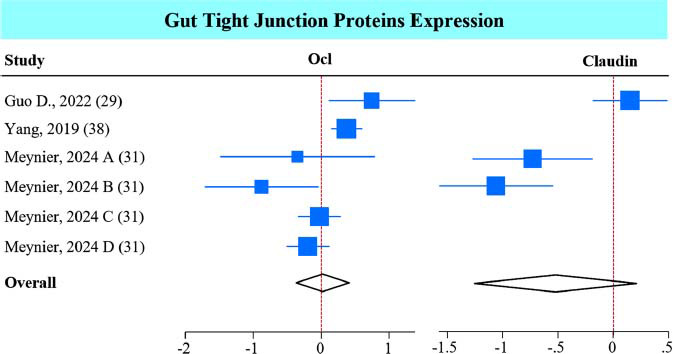
Effect of *A. muciniphila* on tight junction proteins expression in the gut. Forest plot of individual SMD of gut Ocl and claudin 1 expression in mice receiving *A. muciniphila*. The white diamond indicates a non-statistical difference.

Reductions in serum DAO, a marker of intestinal permeability, suggest that *A. muciniphila* may improve intestinal permeability. This was evaluated by measuring the mRNA expression of tight junction proteins in the gut. Surprisingly, *A. muciniphila* showed no effect on occludin (Ocl) expression and reduced claudin 1, which did not reach significance (*P* = 0.16) (Fig. **8**). However, the sub-group analysis revealed that live *A. muciniphila* significantly increased Ocl, while the non-live interventions reduced claudin 1 (Figs. **S9A** and **B**).

### 3.8 *A. muciniphila* and Gut Microbiome

*A. muciniphila*'s role in mental health is largely mediated through the microbiome-gut-brain axis by facilitating neurotransmitter production in the gut and modulating central nervous system signals. Due to the ecological dynamics within the gut microbiome, administering specific bacteria induces significant shifts in microbial composition and interactions, which in turn influences signaling *via* the gut-brain axis. Therefore, understanding the changes in the overall gut microbiome following *A. muciniphila* intervention and deciphering the crosstalk between the altered microbiome and the host is crucial to uncovering the mechanisms underlying *A. muciniphila*'s effects on mental health. To assess these changes, we analyzed three studies in which microbiome data was available, as we previously reported [15]. Two studies focused on depressive disorders and one on other psychiatric disorders, all utilizing live bacterium administration (Table **1**).

**Fig. (9):**
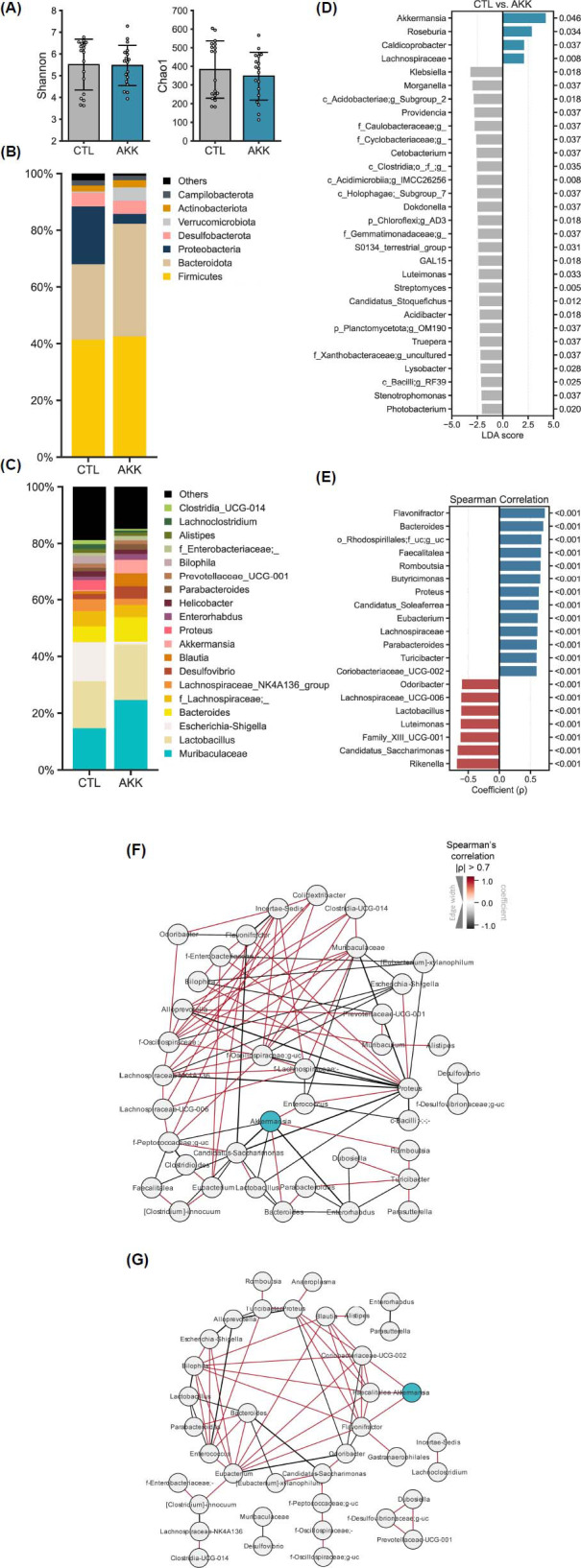
*A. muciniphila* remodels the microbiome. The data was analyzed for microbial alpha diversity, including Shannon and Chao1 (**A**) and microbiome composition at the phylum (**B**) and genus (**C**) levels. Differential abundance analysis results with LEfSe (LDA>2.0, *P*-value < 0.05) (**D**). Significantly correlated genus with *Akkermansia* (Spearman correlation coefficient (ρ) > 0.6) (**E**). Distinct differences between genera in CTL (**F**) and AKK (**G**) groups were observed in correlational networks. Each node in (**F** and **G**) represents one genus, and only significant links between genera are shown (Spearman coefficient (ρ) > 0.75, Benjamini-Hochberg corrected *P*-value < 0.05). The figures were generated using Python (version 3.8.16) package.

Microbial diversity was evaluated using Shannon and Chao1 metrics. Although there were no significant differences between controls (CTL) and *A. muciniphila*-administrated (AKK) groups, the Chao1 diversity of the AKK group was marginally lower than that of the CTL group, suggesting a slight reduction in richness in the AKK group (Fig. **9A**). Subsequently, we compared the composition of microbes at the phylum and genus levels between controls and AKK groups. At the phylum level, three major phyla-Firmicutes, Bacteroidota, and Proteobacteria-accounted for more than 80% of the total bacterial composition in both the CTL and AKK groups. Among them, Verrucomicrobiota, to which *Akkermansia* belongs, and Bacteroidota were more abundant in the AKK group, while Proteobacteria was significantly higher in the CTL group (Fig. **9B**). Specifically, *Akkermansia*, *Roseburia*, *Caldicoprobacter*, and *Lachnospiraceae* were significantly increased in the AKK mice. In contrast, various genera within the Proteobacteria phylum, including *Klebsiella*, *Morganella*, *Providencia*, *Dokdonella*, *Luteimonas*, *Stenotrophomonas*, *Photobacterium*, and *Lysobacter*, were enriched in the CTL mice. Additionally, *Streptomyces* and *Acidimicrobiia*, belonging to Actinobacteria, were more abundant in the CTL mice (Figs. **9C**, **D**).

Next, correlational and network analyses were conducted to explore possible functional associations between *A. muciniphila* and other microbes and to evaluate how *A. muciniphila* treatment alters the microbial ecological niche in the gut (Figs. **9E**-**G**). Correlational analysis revealed that *A. muciniphila* positively correlated with several SCFA-producing bacteria, including *Butyricimonas*, *Eubacterium*, *Lachnospiraceae*, and *Faecalitalea*, as well as with *Flavonifractor*, a flavonoid-degrading bacterium. Conversely, *Candidatus Saccharimonas*, *Odoribacter*, and *Lactobacillus* negatively correlated with *A. muciniphila* (Fig. **9E**). Network analysis, performed separately for the CTL and AKK groups, revealed distinct microbial associations following *A. muciniphila* administration. Interestingly, *A. muciniphila* formed more significant networks with other microbes in the CTL group than in the AKK group. In the CTL group, *A. muciniphila* exhibited co-occurrence associations with *Romboutsia*, *Bacteroides*, and *Enterococcus*, and mutually exclusive associations with an uncultured genus in the *Peptococcaceae* family, *Candidatus Saccharimonas*, *Lactobacillus*, and *Enterorhabdus*. In contrast, in the AKK group, *A. muciniphila* demonstrated positive correlations with *Coriobacteriaceae UCG-002*, *Faecalitalea*, and *Flavonifractor*. Notably, *Faecalitalea* and *Flavonifractor*, which showed mutually exclusive associations with *Candidatus Saccharimonas* in the CTL group, were potentially suppressed by *Candidatus Saccharimonas* but were replenished with the help of *A. muciniphila* in the AKK group (Figs. **9F**, **G**).

## 4 DISCUSSION

The findings of this meta-analysis provide compelling evidence for the positive impact of *A. muciniphila*, its outer membrane protein (Amuc_1100), and EVs on behavioral outcomes in mouse models of depression, anxiety, and stress. The results show that *A. muciniphila* produced significant improvements in several conditions, including alcohol abuse, antibiotic-induced anxiety and depression, chronic stress, HFD-induced memory impairment, TBI, sleep deprivation, neonatal maternal separation, IBD, and Alzheimer’s disease. Fig. (**10**) summarizes the behavioral outcomes assessed in the included studies and proposed mechanisms by which *A. muciniphila* improves brain function. *A. muciniphila* supplementation ameliorated anxiety-like symptoms measured in the OFT, LDB, and EPM tests and improved memory, which was evaluated using the NOR, Y-maze, and Barnes circular maze tests. It also reduced depression-like behavior by reducing the freezing time in the TST and FST tests. Several mechanisms by which *A. muciniphila* improved these outcomes were explored, which implicated factors such as the expression of serotonin and neuronal plasticity-related molecules, inflammation, intestinal epithelial permeability, and microbiome composition.

**Fig. (10):**
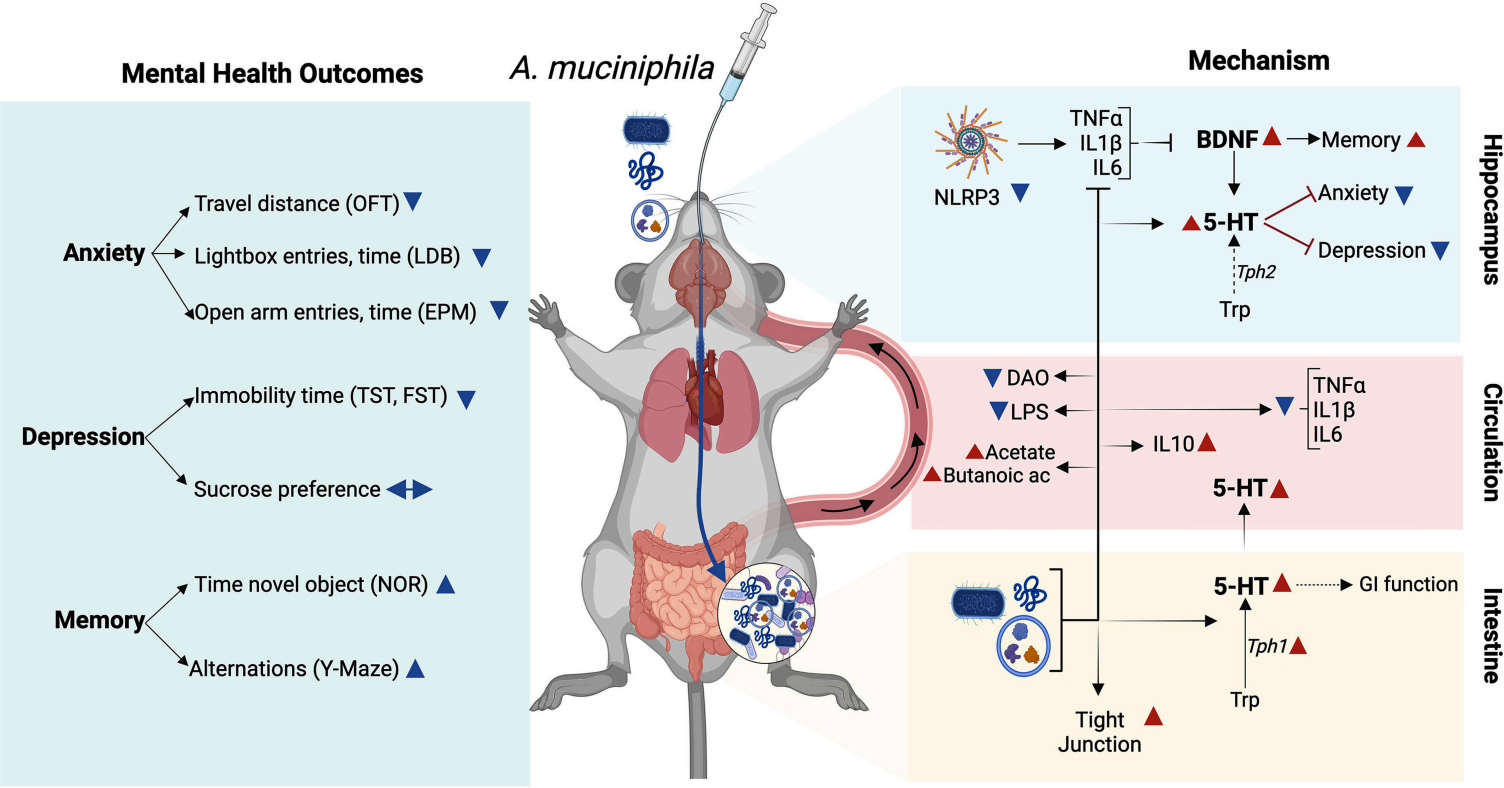
Model for the proposed mechanism by which *A. muciniphila* improved behavioral outcomes. Consumption of *A. muciniphila* and its by-products, such as EVs and Amuc-1100, can lead to improvements in neuropsychiatric function. *A. muciniphila* increases the activity of Tph1 in the gut, leading to an increase in serotonin levels, which plays a role in improving gastrointestinal (GI) function. Additionally, *A. muciniphila* affects the levels of gut-tight junction proteins and enhances the integrity of the gut epithelium, thereby preventing the access of toxic molecules (*e.g*., LPS) to blood circulation. Furthermore, *A. muciniphila* increases the abundance of SCFA-producing bacteria in the gut. By reducing LPS levels and increasing SCFAs in circulation, *A. muciniphila* exerts an anti-inflammatory effect. Inhibiting inflammatory markers in circulation can positively affect neural function by inhibiting neuroinflammation. In the hippocampus, *A. muciniphila* increases Tph2 activity, leading to elevated serotonin levels that reduce behavioral manifestations of anxiety and depression. Additionally, by reducing NLRP3 activity in the hippocampus, *A. muciniphila* reduces neuroinflammation, thereby improving memory. Red and blue arrowheads indicate increased and decreased expression, respectively. The figure was created by BioRender.

*A. muciniphila* elevated serotonin levels in the hippocampus, serum, and gut (Fig. **10**). In the gut, it increased tight junction gene expression, while in circulation, it reduced biomarkers of inflammation (TNFα, IL1β, and IL6) and intestinal permeability (LPS and DAO) while increasing the anti-inflammatory cytokine IL10 and the SCFAs acetate and butanoic acid. In the brain, reduced inflammation was associated with reduced expression of NLRP3, leading to IL1β downregulation. The upregulation of BDNF may explain improved memory outcomes, while elevated serotonin may explain the reduction in symptoms of anxiety and depression.

**Fig. (11):**
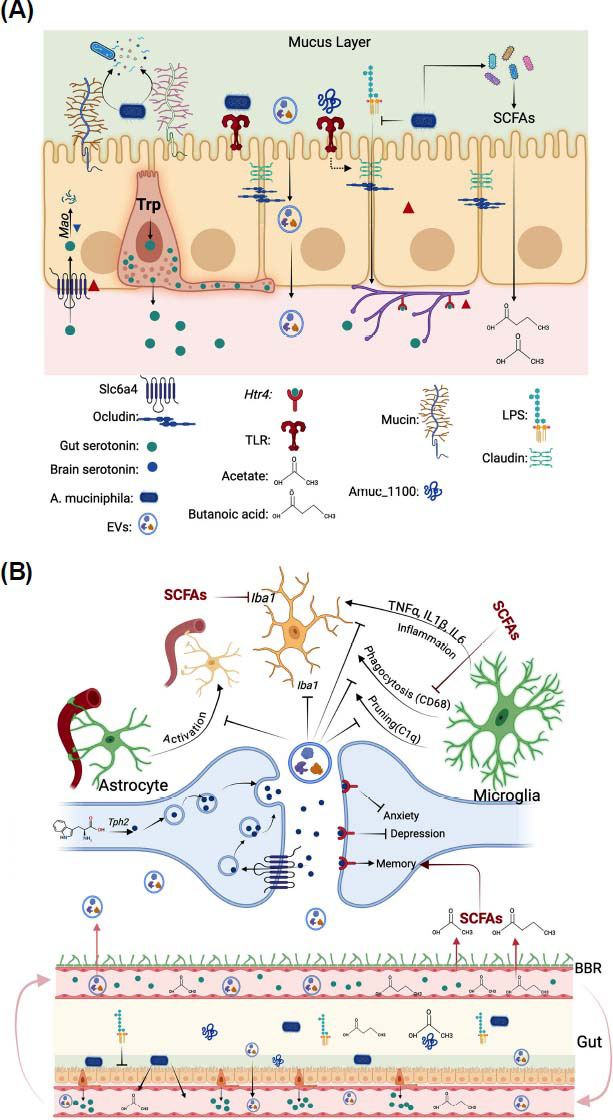
Model for the proposed mechanism by which *A. muciniphila* regulates intestinal and brain function. *A. muciniphila* increases the number of short-chain fatty acid (SCFA)-producing bacteria by degrading mucin and providing energy for these bacteria. The produced SCFAs can be absorbed and transported to the brain, where they reduce microglia phagocytic activity by inhibiting Iba1 and CD68 in the hippocampus. Additionally, *A. muciniphila* influences the expression of gut-tight junction proteins, preventing the leakage of LPS and other toxic molecules into the circulation, thereby controlling systemic inflammation. EVs produced by *A. muciniphila* can pass through the gut epithelium, enter circulation, and cross the blood-brain barrier (BBB) (**A**). In the hippocampus, these EVs inhibit C1q, a marker of microglial pruning and synapse engulfment, as well as phagocytosis, inflammation, Iba1, and astrocyte activity (**B**). The figure was created by BioRender.

Serotonin is produced in the gut (Fig. **11A**) and the brain (Fig. **11B**). Most of the serotonin in the body is found in the gut, being secreted into circulation by enterochromaffin cells (ECs) in the intestinal epithelium. This serotonin pool regulates gastrointestinal function and contributes to serum serotonin (Fig. **11A**). At the protein level, serotonin is regulated by protein synthesis and degradation. Tryptophan is a precursor in two closely related metabolic pathways: serotonin synthesis and the kynurenine pathway, which are regulated by the rate-limiting enzymes TPH1 (TPH1 in the gut and TPH2 in neurons) and IDO, respectively [52]. Serotonin degradation is mediated by the monoamine oxidase (MAO), producing metabolites that are excreted in the urine. Serotonin is transported through the serotonin transporter SERT, which is encoded by the Slc6a4 gene, representing the main mechanism for serotonin reuptake. Lastly, serotonin acts by binding to G-protein-couple serotonin transporters (Htrs). Regarding these genes, several studies showed increased serotonin and Tph1 gene expression in the gut, which is crucial for serotonin biosynthesis [35, 53]. Other genes were also examined; however, they were only examined in one study. For example, Yaghoubfar *et al.* [35] saw increased colonic expression of Slc6a4 and Htr4 and reduced MAO expression (Fig. **11A**).

Slc6a4 is expressed in intestinal epithelial cells and mediates the reuptake of serotonin. Once inside the cells, serotonin can be degraded by the enzyme MAO. Serotonin transporters, such as Htr4, are found in gut serotonergic neurons, which mediate gut motility [54]. This study also used Caco-2 cells to assess serotonergic genes. *A. muciniphila* and EVs increased Slc6a4 expression, and only EVs upregulated serotonin and TPH1 in CaCo_2_ cells. The enzyme DAO, important for metabolizing histamine and maintaining intestinal barrier integrity, shows elevated levels when the barrier is compromised [41], but treatment with *A. muciniphila* restores barrier function.

*A. muciniphila* increased serotonin levels in the brain (Fig. **11B**). This effect was associated with reduced inflammatory markers, including TNFα, IL1β and IL6. Furthermore, *A. muciniphila* can inhibit stress-induced microglial overactivation, reduce Iba1 expression, and prevent synaptic damage in the hippocampus [40, 55]. As with the gut, other markers were also measured in the brain, but only in one study. For example, Li *et al.* [40] reported that sleep deprivation upregulated C1q, a marker of microglia pruning and synapse engulfment, and CD68, a lysosomal marker associated with phagocytic activity. Along with the upregulation of Iba1 observed in this study, these findings indicate that sleep deprivation profoundly impairs microglial function. However, all these markers were reduced following *A. muciniphila* intervention.

Beyond its effects on serotonin levels, *A. muciniphila* also affected other key molecules in the HIPP and serum crucial for neural function. BDNF is a critical growth factor involved in neural development, regeneration, synaptic plasticity, and neurogenesis, particularly in brain regions like the HIPP, which are linked to mood disorders. Reduced BDNF is associated with depression, while increased levels are necessary for antidepressant effects [56, 57]. *A. muciniphila* has been shown to elevate BDNF mRNA expression in the HIPP, potentially improving neuronal connectivity and alleviating depression [2, 33]. Additionally, GRs in the HIPP are activated by stress-induced glucocorticoids, leading to negative effects such as impaired neurogenesis, which can contribute to depression; however, treatment with Amuc_1100 reduces GR levels in chronic stress models [36]. CREB1, a transcription factor regulating depression-related genes, is influenced by stress and inflammatory responses [58-61], with Amuc_1100Δ80 potentially modulating the 5-HTR1A-CREB-BDNF pathway *via* toll-like receptor 2 (TLR2) interactions [36].

Li *et al.* [40] also showed that sleep deprivation reduced the expression of synaptic proteins, including the vesicular glutamate transporter (Vglut1), the postsynaptic density protein 95 (PSD-95), a scaffolding protein in excitatory neurons, and synaptophysin (SYP), an integral protein of presynaptic vesicles. The level of these proteins was also restored by *A. muciniphila,* suggesting that the intervention prevented synapse loss due to sleep deprivation. Interestingly, this study also measured SCFAs, finding that acetate and butanoic acid were reduced by sleep deprivation and restored by *A. muciniphila*. Furthermore, treatment of sleep-deprived mice with acetate and butanoic acid reduced Iba and CD68 in microglia and increased Vglut1 and PSD-95SYP in the HIPP of mice. Regarding behavior, a positive correlation was found between acetate and butanoic acid levels and exploration time in the NOR test.

Similarly, Yang, *et al.* [38] also explored mechanisms that were not measured in the other studies. The authors used HFD (60%kcal of fat) to induce cognitive impairment in young mice. HFD reduced the amplitude of the miniature excitatory postsynaptic current (mEPSC), showing no effects on the frequency or amplitude of the miniature inhibitory postsynaptic current (mIPSC) in hippocampal CA1 neurons. HFD also altered long-term potentiation (LTP) in the HIPP, reduced marker of proliferation, neuronal dendrite length in the dentate gyrus, and expression of the AMPAR receptor GLuA1 and GluA2. In the gut, HFD promoted leaky gut and endotoxemia. *A. muciniphila* restored the levels of these molecules, improving neuronal plasticity and cognitive impairment. The authors further demonstrated that LPS injection causes microglia activation, neuroinflammation in the HIPP, and cognitive impairment. Blockade of TLR4 receptor reduced LPS-induced effects. Regarding BDNF signaling, Sun *et al.* [34] saw an increase in tropomyosin receptor kinase B (TrkB), a receptor tyrosine kinase that binds BDNF, and the downstream target c-Fos in the HIPP with both *A. muciniphila* and Amuc_1100. The intervention also restored astrocyte activation, measured by glial fibrillary acidic protein (GFAP).

Most of the sub-group analyses showed significant effects of both live and non-live interventions; however, some factors such as time in lightbox (anxiety test), FST (depression test), serum and hippocampus serotonin level, hippocampus GR level, and hippocampus TNFα level were significantly affected by non-live interventions.

Our findings indicate a significant effect of *A. muciniphila* on reducing biomarkers of inflammation in both serum and HIPP. The significant decrease in neuroinflammatory markers, including TNFα, IL1β, and IL6 in the HIPP, highlights another potential pathway through which *A. muciniphila* exerts its effects. Chronic neuroinflammation has been associated with the pathophysiology of depression and anxiety [62-64], and reducing inflammation in the brain may help alleviate these conditions. Haapakoski *et al.* [65] found significant associations between depression and increased levels of peripheral inflammatory markers in cerebrospinal fluid (CSF), including C-reactive protein (CRP) and IL6. Likewise, Su *et al.* [66] identified a connection between interferon-α (IFNα) in the brain parenchyma and the onset of depression. Moreover, in mouse models displaying depressive-like behaviors, elevated expression of genes related to inflammation and injury repair was noted in the HIPP [67]. *A. muciniphila* downregulates pro-inflammatory pathways in the central nervous system (CNS) and the bloodstream, notably by suppressing systemic inflammatory biomarkers, including IL6 [68]. Overproduction of IL6 can reduce neurogenesis and impair neurotransmission in brain regions such as the HIPP and prefrontal cortex, both of which are crucial for cognitive function. Zhu *et al.* [68] showed that *A. muciniphila* treatment significantly downregulated the expression of genes involved in the T helper (Th17) cell differentiation induced by the IL6 pathway. Moreover, by suppressing Th17 cell activity and diminishing microglial hyperactivity in the brain, *A. muciniphila* lowers the release of pro-inflammatory cytokines, improving cognitive function. Both *A. muciniphila* and its protein Amuc_1100 effectively suppress the recruitment and activation of CD16/32+ M1 macrophages by decreasing the number of cytotoxic T-lymphocytes [69]. M1 macrophages are known for their rapid inflammatory response to infections and tissue damage, so reducing their numbers can help mitigate inflammatory symptoms throughout the body [70, 71]. Chen *et al.* [39] found that administering *A. muciniphila* orally led to decreased microglia activation and Nlrp3 inflammasome activity in the brain, which improved outcomes related to neuroinflammation and nerve injury following TBI. Neuroinflammation impairs neurogenesis in the HIPP, negatively impacting cognition and memory, resulting in symptoms such as impaired working memory, inattention, and heightened negative cognitive biases [72, 73]. Therefore, the improvement in memory status may partly result from the positive effects of *A. muciniphila* on neuroinflammation.

The analysis of the data related to gut barrier function showed a non-significant effect of *A. muciniphila* on tight junction protein expression when all studies were combined. However, sub-group analysis shows that improvement in these markers depends on the intervention (live *vs.* non-live bacterium). The overall non-significant results might stem from the limited number of included studies. Our recent meta-analysis on cardiometabolic outcomes found that *A. muciniphila* supplementation significantly improved tight junction protein expression in the gut [15]. Improved gut barrier function is significant to neuropsychiatric disorders because disruption of this barrier is linked to anxiety, depression, and autism spectrum disorders (ASD). In fact, *A. muciniphila* supplementation can significantly increase the mucus layer thickness by increasing mucin 2 expression and the number of goblet cells [32]. As this bacterium resides in the mucus layer, it can activate the Wnt/β-catenin signaling pathway to repair and maintain the gut mucosal barrier [74]. Additionally, Amuc_1100 activates TLR2-mediated signaling pathways, increasing the expression of tight junction proteins [9]. Similarly, administration of EVs increased Ocl expression, improving gut barrier function [75].

The microbiome analyses revealed a strong correlation between *A. muciniphila* and SCFA-producing bacteria. *A. muciniphila* has been shown to enhance intestinal homeostasis by promoting intestinal stem cell-mediated epithelial development following mucin degradation [76, 77]. Mucin degradation by *A. muciniphila* has been found to increase the availability of health-promoting oligosaccharides, including O-linked glycans and SCFAs, within the gut [78]. This alters the gut microbial niches and modulates the abundance of specific microbes that consume and utilize these metabolic products for their metabolism [79]. Studies have demonstrated that mucin-derived O-linked glycans increase the intestinal population of butyrate-producing bacteria while elevating the levels of butyrate and acetate in the cecum of mice [79]. One such butyrate-producing bacterium, *Faecalitalea*, showed a positive correlation with *A. muciniphila* in our analysis [80]. In prior research, *Faecalitalea* showed a negative correlation with brain aging factors [81] and exerted an indirect mediating effect on impulsivity, reducing impulsivity in methamphetamine abusers with higher *Faecalitalzea* abundance [82]. Although direct evidence linking *Faecalitalea* to brain function and mental health is limited, its role in producing butyrate—a compound known for its neuroprotective functions and contribution to brain homeostasis—suggests that *Faecalitalea* may be associated with the positive effects of *A. muciniphila* on mental health through its metabolic products [83].

The capacity of *A. muciniphila* to increase butyrate can have several consequences for key physiological processes in mood and anxiety conditions. First, butyrate inhibits the nuclear translocation of GRα, preventing the effects of stress and circadian cortisol/corticosteroids [84], including upregulation of tryptophan 2,3-dioxygenase (TDO), which, like IDO, converts tryptophan to kynurenine [85]. Second, butyrate regulates epigenetic modifications by inhibiting the activity of histone deacetylases (HDACs) [86]. HDACs regulate excitatory amino acid transporters, like excitatory amino acid transporter (EEAAT) 2 [87], that show alterations in mood/anxiety disorders [88]. Third, butyrate can promote mitochondrial function, at least in part, by activation of the pyruvate dehydrogenase complex implicated in melatonin synthesis [89]. Fourth, butyrate is an important metabolic substrate not only for intestinal epithelial cells but also for astrocytes [90], with recent work indicating the important role of astrocytes in the pathophysiology of depression *via* the mitochondrial melatonergic pathway [91].

Another bacterium associated with *A. muciniphila* is *Flavonifractor*, which has been found to exhibit ambivalent health effects. While oral administration of *Flavonifractor* has been shown to attenuate inflammatory responses and play a protective role in cardiovascular health [92, 93], a high prevalence of this bacterium has been observed in affective disorders, bipolar disorder, and major depressive disorder [94-96]. Most studies reporting negative effects of *Flavonifractor* attribute these effects to degradation of beneficial flavonoids during metabolism, which may be the primary reason for its detrimental impact. However, *Flavonifractor* is also an SCFA producer, including propionate and valerate [97]. Considering that flavonoids have been found to enhance the growth of Proteobacteria in the gut in some studies [98], the reduction in Proteobacteria by *A. muciniphila* may possibly be due to decreased flavonoid levels resulting from increased *Flavonifractor* activity. Nevertheless, further studies would be needed to validate and elucidate the specific role of *Flavonifractor* in brain function.

In summary, the findings of this study showed that consumption of *A. muciniphila* could ameliorate behavioral symptoms associated with depression, anxiety, and stress. *A. muciniphila* interventions (live, heat-killed, Amuc_1100, and EVs) alleviate symptoms of neuropsychiatric disorders through several mechanisms, including reduced gut, systemic, and brain inflammation, gut microbiota remodeling, and improvement of the gut mucosal barrier. The observed effects on neurobehavioral outcomes and biochemical markers suggest that *A. muciniphila* could be developed as a novel therapeutic intervention for neuropsychiatric disorders. Additionally, the observed reduced serum cortisol levels by *A. muciniphila* indicate a modulation of the hypothalamic-pituitary-adrenal (HPA) axis, which is involved in stress responses. Dysregulation of this system is often implicated in anxiety and depressive disorders. The attenuation of cortisol levels suggests that *A. muciniphila* may help normalize the stress response, potentially offering a protective effect against stress-related disorders.

## 5 LIMITATIONS

This meta-analysis has several limitations, including the heterogeneity in study designs. For example, differences in mouse strains, models of induced stress or depression, dosages, and duration of *A. muciniphila* administration may introduce variability in the outcomes. Additionally, the exclusive focus on preclinical models limits the generalizability of the findings to human populations. While animal models provide valuable insights into biological mechanisms, they do not fully replicate the complexity of human neuropsychiatric conditions. Furthermore, the relatively small number of included studies may limit the statistical power of the analysis, particularly for detecting small effect sizes or non-significant trends. Future meta-analyses with a larger pool of studies and more consistent methodologies would strengthen the evidence base.

## CONCLUSION

In conclusion, this systematic review and meta-analysis provide strong preliminary evidence that *A. muciniphila* can ameliorate behavioral symptoms associated with depression, anxiety, and stress in mouse models, likely through modulation of the microbiota-gut-brain axis, serotonergic pathways, HPA axis regulation, and reduction of neuroinflammation. These findings suggest that *A. muciniphila* could serve as a novel therapeutic intervention for mental health disorders, offering a potential alternative to traditional pharmacological treatments. The prospect of using probiotics to modulate mental health represents an exciting frontier in neuroscience and microbiome research, with significant implications for developing new, biologically based therapies.

## Figures and Tables

**Table 1:** Studies with publicly available data for microbiome analysis.

**Study**	**Disease**	**Treatment**	**No. of Samples**
Guo *et al*. [29]	Depressive disorders	Live *Akkermansia*	CTL (n=4), AKK (n=4)
Chen *et al*. [32]	Psychological disorder	Live *Akkermansia*	CTL (n=9), AKK (n=9)
Ding *et al*. [33]	Depressive-like behavior	Live *Akkermansia*	CTL (n=5), AKK (n=5)

**Note**: Control (CTL): 18 samples, *Akkermansia* (AKK) 18 samples.

## LIST OF ABBREVIATIONS

ASD: Autism Spectrum Disorders
BDNF: Brain-derived Neurotrophic Factor
CRP: C-reactive Protein
CNS: Central Nervous System
CSF: Cerebrospinal Fluid
CRS: Chronic Restraint Stress
CUMS: Chronic Unpredictable Mild Stress
DAO: Diamine Oxidase
EPM: Elevated Plus Maze
FST: Forced Swim Test
HFD: High-fat Diet
IBD: Inflammatory Bowel Disease
LPS: Lipopolysaccharide
NMS: Neonatal Maternal Separation
OFT: Open Field Test
PTSD: Post-traumatic Stress Disorder
SCFAs: Short-chain Fatty Acid
TST: Tail Suspension Test
TBI: Traumatic Brain Injury

## STANDARDS OF REPORTING

PRISMA guidelines and methodology were followed.

## SUPPLEMENTARY MATERIAL

PRISMA checklist is available as supplementary material on the publisher’s website along with the published article. Supplementary material is available on the publisher's website along with the published article.
