# Supplementary material for: The Impact of Akkermansia muciniphila on Mouse Models of Depression, Anxiety, and Stress: A Systematic Review and Meta-Analysis
Source: Curr Neuropharmacol. 2025 Mar 18;23(11):1423–41. doi: 10.2174/011570159X360149250225041829 (PMC12606656; doi:10.2174/011570159X360149250225041829)
Supplement: Supplementary file 1 — PRISMA checklist is available as supplementary material on the publisher’s website along with the published article. Supplementary material is available on the publisher's website along with the published article. [file CN-23-11-1423_SD1.pdf]

Supplementary Material

The Impact of *Akkermansia muciniphila* on Mouse Models of Depression, Anxiety, and Stress: A Systematic Review and Meta-Analysis

Leila Khalili[1], Gwoncheol Park[1], Ravinder Nagpal[1], Pradeep Bhide[2] and Gloria Salazar[1,*]

[1]Department of Health, Nutrition, and Food Sciences, Florida State University, Tallahassee, FL 32306, USA; [2]Institute for Pediatric Rare Diseases and Department of Biomedical Sciences, Florida State University, College of Medicine, Tallahassee, FL 32306, USA

1875-6190/25 $65.00+.00 **© 2025 Bentham Science Publishers**

Table S1. Egger and Beg test results of the analyzed factors.

Marker	Egger	Beg
**OFT**	0.0003	0.1611
**Memory**	0.6344	0.9015
**Learning time**	0.0000	1.0000
**FST**	0.7768	0.3675
**TST**	0.3442	0.3481
**Entries in light box (LDB)**	0.3335	1.0000
**Time in light box (LDB)**	0.0131	0.0354
**EPM**	0.0124	0.2207
**Serum Serotonin**	0.6090	0.8580
**HIPP Serotonin**	0.0000	0.1753
**Colon Serotonin**	0.0805	1.0000
**Serum Corticosterone**	0.2107	1.0000
**HIPP BDNF**	0.8560	0.7639
**HIPP Iba1**	0.0199	0.3082
**HIPP GR**	0.6465	1.0000
**Gut TPH1**	0.9204	0.7071
**Hippocampus TNFα**	0.0000	0.7071
**Hippocampus IL1β**	0.4087	1.0000
**Hippocampus IL6**	0.9756	1.0000
**Ocl expression**	0.3766	0.5661
**Claudin expression**	0.0047	1.0000

Table S2. Characteristics of included studies.

N	Author and Year	Disorder/ Condition	Strain	Sex	Age	*Akk* Strain	Diet	Intervention	Overall Conclusion
1	Guo, D., 2022 (29)	Alcohol/LPS depression model (mALSP)	C57BL/6	F	7-8 w	NR	Maintenance diet SPF	*A. muciniphila* 1.5×10^8 cfu every 3 days for 2 weeks	*Akk* reduced neuroinflammation (TNFα, IL1β in hippocampus), serum TNFα, IL6 and LPS, increased 5-HT and BDNF, and reduced IDO and microglial activity in the HIPP. *Akk* improved depression symptoms increasing total travel time and reducing time in the periphery in OFT and immobility time in the FST and TST. *Akk* increased gut occludin expression.
2	Sun, Y., 2023 (34)	Antibiotic-induced anxiety and depression model	C57BL/6	M	6 w	NR	Sterile chow SPF	*A. muciniphila* 1.5×10^9 cfu/200 µl daily for 2 weeks	*Akk* increased entries and time in the light box in the LDB test and decreased immobility time in the TST and FST. *Akk* increased Firmicutes, bacilli and clostridia in gut. *Akk* increased 5-HT, BDNF, TrkB and c-Fos and reduced GFAP and GR in HIPP and reduced serum Cort.
							Normal chow food	Amuc_1100 (outer membrane protein) 100 µg/200 µl daily for 2 weeks	Amuc increased entries and time in the light box in the LDB test and increase entries and time at the center in the OFT test, and total travel time, increased OE/TE in the EPM, and decreased immobility time in the TST and FST. Amuc increased *Firmicutes*, *Bacilli*, *Clostridia* and *Oscillospira-ceae* in the gut. Amuc increased HIPP and cortex BDNF, TrkB and c-Fos and reduced GFAP, increased serum and HIPP 5-HT and reduced HIPP GR.
3	Ding, Y., 2021 (33)	Chronic restraint stress depression model	C57BL/6	M	6-8 w	ATCC® BAA-835™	Standard diet	*A. muciniphila* $1{\times}10^8$cfu/mL daily for 3 w	*Akk* increased total travel distance in the OFT and reduced immobility time in the TST and FST test. *Akk* reduced serum corticosterone, increased serum dopamine and increased HIPP BDNF. Serum 5-HT also trended upwards by using *Akk*. *Akk* restored food intake and BW of mice to control levels. *Akk* decreased *Helicobacter*, *Candidatus_Saccharimonas*, *Eubacterium_ branchy*_gr and *Lachnoclostridium in gut. Akk* increased serum βAMH and edaravone (Eda) to relive depression.
						CICC 23,133		*L. plantarum-* $5{\times}10^8$cfu/mL	*L. plantarum* increased total travel distance in the OFT and reduced immobility time in the TST and FST test
						CICC 23,133		*L. plantarum* $5{\times}10^9$cfu/mL	*L. plantarum* increased total travel distance in the OFT and reduced immobility time in the TST and FST test.
								βAMH 10 mg/mouse	βAMH increased total travel distance in the OFT and

N	Author and Year	Disorder/ Condition	Strain	Sex	Age	*Akk* Strain	Diet	Intervention	Overall Conclusion
								3d	reduced immobility time in the TST, reduced serum Cort and increased serum 5-HT and dopamine and HIPP BDNF (ns).
								Eda 10 mg/mouse 7d	Eda increased total travel distance (OFT) and reduced immobility time (TST), serum Cort, and increased serum dopamine (ns) and 5-HT and HIP BDNF (ns).
4	Yaghoubfar, R., 2020 (35)	Healthy	C57BL/6J	M	8 w	ATCC BAA-835	Standard diet	*A. muciniphila* 10^9 cfu in PBS daily for 4 w	*Akk* increased 5-TH in colon and HIPP and reduced serum 5-HT. In the colon, *Akk* increased Tph1, Slc6a4 and Htr4 and reduced Htr2B, Htr3B and Htr7. In the HIPP *Akk* increased Tph2 and reduced Slc6a4, Mao, Htr1A, Htr2A and Htr5 and 6. *Akk* increased IL10 in colon.
		Healthy	C57BL/6J	M	8 w	ATCC BAA-835	Standard diet	EVs of *A. muciniphila* 10 µg daily for 4 w	EVs increased 5-TH in colon (better than *Akk*) and HIPP and reduced serum 5-HT. In the colon, EVs increased Tph1, Slc6a4 and Htr4 and reduced Mao Htr2B, Htr3B and Htr7. In the HIP EVs increased Tph2, and reduced Slc6a4, Mao and Htr1A, Htr2A and Htr5 and 6. EVs had a stronger effect than *Akk* in these markers. EVs Increased IL10 and reduced TNFα in colon.
5	Cheng, R., 2022 (36)	Chronic unpredictable mild stress (CUMS)	C57BL/6	M	5-6 w	NR	Standard diet	Amuc_1100 80 µg daily for 4 w	Both interventions reduced immobility time in the FST and TST and entries in the open arm in EPM test (ns). Only Amuc $1100^{\Delta80}$ increased total distance in the OFT. Amuc $1100^{\Delta80}$ was better in increasing entries and time in the light box in the LDB test. Both interventions increased 5-HT and BDNF in HIPP and serum 5-HT. In the ilium both interventions reduced IL6, TNFα and IL1β. Both interventions reduced IL1β and TNFα in serum and frontal cortex. Both interventions reduced HIPP GR and only Amuc $1100^{\Delta80}$ reduced Cort.
							Standard diet	Amuc_$1100^{\Delta80}$ 80 µg daily for 4 w	
6	Cheng, R., 2021 (37)	CUMS	C57BL/6	M	5-6 w	NR	Standard diet	Amuc_1100 80 µg daily for 6 w	Amuc increased serum and colon 5-HT and colon Tph1, reduced IL6, IL1β and TNFα in HIPP. Amuc increased time in light box (ns) and reduced immobility time in TST (ns).
7	Yang, Y., 2019 (38)	Learning and memory impairment in HFD-induced obesity early in	C57BL/6J	M	Juvenile (P21)	ATCC BAA845	HFD 60% Kcal Fat	*A. muciniphila* 5×10^9 cfu/ml every 12 hours for 28 days	Live *Akk*, increased freezing time% in CFC and reduced errors in BCM test, increased neuronal proliferation, dendrite length and synaptic density in the DG, increased mEPSC amplitude and frequency and GluA1 and 2 in the HIPP, reduced Iba1, IL1β, IL6 and TNFα

N	Author and Year	Disorder/ Condition	Strain	Sex	Age	*Akk* Strain	Diet	Intervention	Overall Conclusion
		life							in the HIPP and increased TJ proteins in the colon reducing serum LPS. Blockade of TLR4 prevented HIPP-dependent learning and memory deficits in HFD-fed mice.
8	Chen, Y., 2023 (39)	Traumatic brain injury (TBI)	C57BL/6N	M	8 w	NR	Lab Mice Diet	*A. muciniphila* 1×10^9 cfu daily for 3 days	*Akk* reduces neuroinflammation in the cortex (Nlrp3, casp1, IL1β, TNFα) and serum DAO and D-LA induced by TBI. *Akk* reduced Iba1, and serum DAO and D-LA (indicators of intestinal barrier damage).
9	Chen, T., 2021 (32)	Chronic Restraint Stress (CRS)	C57BL/6N	M	nm	DMS 22959	Standard diet	*A. muciniphila* 1×10^8 cfu daily for 14 days	CRS reduced Akk abundance in the gut. *Akk* increased total travel distance in the OFT and reduced immobility time in the TST and FST. *Akk* increased colon length, Muc2 mRNA, Muc2 positive cells and goblet cells, reduced colon histopathological scores and increased *Ruminiclostridium* in the gut.
10	Guo, H., 2023 (43)	National Institute on Alcohol Abuse and Alcoholism (NIAAA) model	C57BL/6J	M	8 w	ATTC BAA-835	Lieber-DeCarli alcohol liquid diet	*A. muciniphila* 2.5×10^9 cfu daily for 4 w	*Akk* reduced immobility time in FST, increased 5-HT in the prefrontal cortex, with no effect in OFT and FC, or intestinal 5-HT or Tph1. *Akk* reduced SERT and Tph1 in intestine.
		Chronic alcohol gavage model					Increasing alcohol doses once a day for 3 w	*A. muciniphila* 2.5×10^9 cfu daily for 3 w	*Akk* reduced immobility time in TST and FST, and increased sucrose preference (SP)%. *Akk* reduced serum ALT and AST and increased intestinal and prefrontal cortex 5-HT. No effect was found in intestinal Tph1
		Depressive-like behavior induced by CUMS			6 w		NR	*A. muciniphila* 2.5×10^9 cfu daily for 5 w	*Akk* increased swimming time in FST and 5-TH in intestine and prefrontal cortex. *Akk* reduced intestinal Tph1, SERT and c-Fos and increased serotonin in cortex.
11	Li, N., 2023 (40)	Sleep-deprived	C57BL/6J	M	5 w	ATCC BAA-835	Commercial standard food	*A. muciniphila* 2×10^8 cfu 3 days a week for 4 weeks	*Akk* improved memory in the NOR and increased Y-maze alternation. No effect was found on OFT or EPM. *Akk* increased Vglut1, SYP and PSD-95 in the dentate gyrus, reduced Iba1, microglia pruning and synapse engulfment (C1q) and CD68 (lysosomal marker in the HIPP). *Akk* increased serum acetate and butanoic acid, which prevent synapse loss and synapse engulfment induced by sleep deprivation.

N	Author and Year	Disorder/ Condition	Strain	Sex	Age	*Akk* Strain	Diet	Intervention	Overall Conclusion
12	Wang, J., 2021 (42)	Antibiotic-treated mice	C57BL/6	M	6–8 w	ATCC BAA-835	Autoclaved normal chow food	*A. muciniphila* 1.5×10^9 cfu daily gavage for two weeks	Both *Akk* and Amuc_1100 enhanced Tph1 expression while reducing SERT expression in the colon. This led to a significant increase in 5-HT levels in both colon tissue and blood. *Akk* and Amuc_1100 promoted gastrointestinal motility and restored the abundance and diversity of gut microbiota in mice that had been treated with antibiotics.
								Amuc_1100 100 µg daily gavage for two weeks	
13	Wu, F., 2019 (30)	HFD-induced metabolic disorders	C57BL/6	F	8 w	ATCC BAA-835	HFD	*A. muciniphila*10^9 cfu for 10 months	*Akk* reduced body weight gain, improved the spatial memory in the Y-maze test, increased Nissl bodies in neurons of the HIPP, and restored the HFD-inhibited tryptophan metabolism. *Akk* prevented the HFD-induced decrease in serum 5-HT and depression, increased the relative fecal abundance of *Bifidobacterium* and was negatively correlated with the fecal abundance of *Bacteroides*. *Akk* increased the SCFAs, isovaleric acid, propionic acid and acetic acid, production.
14	Meynier, M., 2023 (31)	Neonatal maternal separation (NMS) IBD model	C57BL/6J	M, F	5-6 w	Pasteurized AKK ATCC BAA-835	Commercial standard food	*A. muciniphila* 6×10^8 and $3x10^9$ cfu	*Akk* reduced colonic hyperactivity, increased intestinal barrier function, reduced serum dextran and increased IL22 in the colon.
		Citrobacter rodentium infection PI-IBD model		M, F	5-6 w				*Akk* reduced colonic hyperactivity and claudin 2 at both doses. *Akk* increased entries in open arms and time spent in open arms in the EPM and increased head dips in the hole-board test. *Akk* increased alternations in the Y-maze, time spend in novel object in the NOR and IL22 in the colon.
15	Ou, Z., 2020 (41)	Alzheimer's disease model	APPswe/PS1dE9 (APP/PS1)	M	3 M	Chinese strain	Normal chow diet	*A. muciniphila* 5×10^9 cfu each day for 6 months	*Akk* reduced fasting blood glucose, blood lipid levels, and serum DAO, improved hepatic steatosis and scapular brown fat whitening and alleviated the reduction of colonic mucus cells in APP/PS1 mice. *Akk* shortened the learning time and improved the completion rate in Y-maze tests and increased the time spent at the center of the OFT.
							HFD		

Abbreviations: HIPP: hippocampus; BDNF: Brain-derived neurotrophic factor; GRs: Glucocorticoid receptors; BW: Body weight; HT4R: serotonin 5-HT4 receptor; Tph1 and 2: Tryptophan hydroxylase 1 and 2**;** Mao: Monoamine oxidase**;** DG: dentate gyrus; GluA1 and 2: AMPA receptor subunit 1 and 2; TJ: tight junction; FC: Forced Swim Test; BCM: Barnes circular maze; CFC: Contextual fear conditioning; TrkB: Tropomyosin receptor kinase B; GFAP: Glial fibrillary acidic protein; Cort: corticosterone; βAMH: β-alanyl-methyl-L-histidine; mEPSC: miniature excitatory postsynaptic currents; NR: non-reported; SERT: serotonin reuptake transporter; Vglut1: Vesicular Glutamate Transporter 1; SYP: Synaptophysin; PSD-95: Postsynaptic Density Protein 95; ns: non-significant, HFD: high fat diet

Table S3. Standardized mean difference (SMD) and its 95% confidence interval (CI) of included studies.

Test	Author, year	Intervention	n/group	SMD (95% CI); P-value
**Anxiety**				
LDB (entries in light box)	Cheng, R., 2022 (36)	A (Amuc_1100)	6	2.46 (1.26, 3.66)
		B (Amuc_1100 Δ80)	6	6.54 (4.75, 8.33)
	Sun, Y., 2024 (34)	A (A. muciniphila, live)	6	5.47 (1.65, 9.29)
		B (Amuc_1100)	6	6.64 (2.82, 10.46)
**Overall P-value**				5.00 (2.68, 7.33); <0.001
LDB (time in light box)	Cheng, R., 2022 (36)	A (Amuc_1100)	6	130.77 (82.11, 179.43)
		B (Amuc_1100 Δ80)	6	200.00 (158.58, 241.42)
	Cheng, R., 2021 (37)	A (Amuc_1100)	7	6.92 (-17.56, 31.40)
		B (Amuc_1100)	7	18.87 (7.26, 30.48)
	Sun, Y., 2024 (34)	A (A. muciniphila, live)	6	27.27 (-2.42, 56.96)
		B (Amuc_1100)	6	36.36 (6.67, 66.05)
**Overall P-value**				68.17 (6.99, 129.35); 0.03
EPM (entries in open arm)	Cheng, R., 2022 (36)	A (Amuc_1100)	6	0.92 (0.37, 1.47)
		B (Amuc_1100 Δ80)	6	0.92 (0.23, 1.61)
	Meynier, M., 2024 (31)	C1 (*A. muciniphila*, pasteurized)	12	1.94 (0.87, 3.01)
**Overall P-value**				1.06 (0.66, 1.46); <0.001
EPM (time spent in open arms)	Sun, Y., 2024 (34)	A (A. muciniphila, live)	6	13.33 (1.22, 25.44)
		B (Amuc_1100)	6	9.70 (-7.09, 26.49)
	Meynier, M., 2024 (31)	C2 (*A. muciniphila,* pasteurized)	12	20.32 (6.45, 34.19)
**Overall P-value**				14.84 (6.82, 22.85); <0.001
OFT	Guo, D., 2022 (29)	*A. muciniphila* (live)	5	846.00 (-172.81, 1864.81)
	Guo, H., 2023 A (43)	A (*A. muciniphila,* live)	12	-933.34 (-2102.03, 235.35)
		B (*A. muciniphila,* live)	12	-275.00 (-690.77, 140.77)

Test	Author, year	Intervention	n/group	SMD (95% CI); P-value
		C (*A. muciniphila*, live)	15	1000.00 (-385.90, 2385.90)
	Cheng, R., 2022 (36)	A (Amuc_1100)	6	-2307.69 (-5747.37, 1132.35)
		B (Amuc_1100 Δ80)	6	7692.31 (3549.95, 11834.67)
	Cheng, R., 2021 (37)	A (Amuc_1100)	7	0.30 (-0.31, 0.91)
	Chen, T., 2021 (32)	*A. muciniphila* (live)	6	10345.00 (4610.13, 16079.87)
	Sun, Y., 2024 (34)	A (*A. muciniphila,* live)	6	4098.36 (-445.58, 8642.30)
		B (Amuc_1100)	6	9836.06 (5292.11, 14380.01)
	Ding, Y., 2021 (33)	*A. muciniphila* (live)	6	1417.00 (1034.83, 1799.17)
**Overall P-value**				2306.77 (39.04, 4574.50); 0.05
**Depression**				
FST	Guo, H., 2023 (43)	A (*A. muciniphila*, live)	12	-15.00 (-30.31, 0.31)
		B (*A. muciniphila,* live)	12	-17.50 (-31.36, -3.64)
		C (*A. muciniphila*, live)	15	2.00 (-1.29, 5.29)
	Cheng, R., 2022 (36)	A (Amuc_1100)	6	-12.31 (-14.48, -10.14)
		B (Amuc_1100 Δ80)	6	-13.54 (-15.50, -11.58)
	Cheng, R., 2021 (37)	B (Amuc_1100)	7	-.347 (-16.26, 9.32)
	Chen, T., 2021 (32)	*A. muciniphila* (live)	6	-8.71 (-91.57, 74.15)
**Overall P-value**				-9.26 (-15.74, -2.78); 0.01
Sucrose Preference	Guo, H., 2023 (43)	B (*A. muciniphila,* live)	12	4.40 (1.89, 6.91)
	Wu, F., 2019 (30)	*A. muciniphila* (live)	10	-0.04 (-0.17, 0.09)
**Overall P-value**				2.00 (-2.34, 6.33); 0.37
TST	Guo, D., 2022 (29)	*A. muciniphila* (live)	5	-56.90 (-121.03, 7.23)
	Guo, H., 2023 (43)	B (*A. muciniphila,* live)	12	-8.33 (-19.89, 3.23)
	Cheng, R., 2022 (36)	A (Amuc_1100)	6	-52 (-80.92, -24.86)
		B (Amuc_1100 Δ80)	6	-67.31 (-93.20, -41.42)

Test	Author, year	Intervention	n/group	SMD (95% CI); P-value
	Cheng, R., 2021 (37)	A (Amuc_1100)	7	-21.80 (-60.22, 16.62)
	Chen, T., 2021 (32)	*A. muciniphila* (live)	6	-72.67 (-158.25, 12.91)
	Sun, Y., 2024 (34)	A (A. muciniphila)	8	-55.39 (-88.00, -22.78)
		B (Amuc_1100)	8	-73.85 (-112.23, -35.47)
	Ding, Y., 2021 (33)	*A. muciniphila* (live)	6	-71.42 (-91.22, -51.62)
**Overall P-value**				-50.35 (-69.20, -31.50); <0.001
**Memory and learning**				
Memory (Y-maze and NOR)	Li, N., 2023 (40)	A1 (*A. muciniphila,* live)	16	14.82 (9.69, 19.95)
		A2 (*A. muciniphila,* live)	16	8.73 (4.70, 12.76)
	Meynier, M., 2024 (31)	C1 (*A. muciniphila,* pasteurized)	12	3.23 (-3.04, 9.50)
		C2 (*A. muciniphila,* pasteurized)	12	27.62 (23.99, 31.25)
		C3 (*A. muciniphila,* pasteurized)	12	27.42 (20.37, 34.47)
	Yang, Y., 2019 (38)	A (*A. muciniphila,* live)	8	23.64 (6.52, 40.76)
		B (*A. muciniphila,* live)	8	39.55 (21.20, 57.9)
		C (*A. muciniphila*, live)	8	4.55 (-17.72, 26.82)
**Overall P-value**				18.25 (9.89, 26.62); <0.001
Learning (Y-maze)	Ou, Z., 2020 (41)	A (*A. muciniphila,* live)	8	-200.00 (-300.78, -99.22)
		B (*A. muciniphila,* live)	8	-218.18 (-307.29, -129.07)
	Yang, Y., 2019 (38)	(*A. muciniphila,* live)	8	-40.91 (-60.82, -21.00)
**Overall P-value**				-144.94 (-262.71, -27.16); 0.02
**Neurotransmitters**				
HIPP Serotonin	Guo, D., 2022 (29)	*A. muciniphila* (live)	5	1.87 (-0.91, 4.65)
	Guo, H., 2023 (43)	A (*A. muciniphila,* live)	12	55.82 (29.12, 82.52)
		B (*A. muciniphila,* live)	12	55.82 (26.81, 84.83)
		C (*A. muciniphila*, live)	15	0.10 (0.08, 0.12)

Test	Author, year	Intervention	n/group	SMD (95% CI); P-value
	Yaghoubfar, R., 2020 (35)	A (*A. muciniphila,* live)	10	0.04 (-0.00, 0.08)
		B (EV)	10	0.06 (0.03, 0.09)
	Cheng, R., 2022 (36)	A (Amuc_1100)	3	38.82 (30.93, 46.71)
		B (Amuc_1100 Δ80)	3	38.82 (33.83, 43.81)
	Cheng, R., 2021 (37)	B (Amuc_1100)	3	1.00 (0.44, 1.56)
**Overall P-value**				18.98 (3.61, 34.36); 0.02
Serum Serotonin	Yaghoubfar, R., 2020 (35)	A (*A. muciniphila,* live)	10	-0.07 (-0.09, -0.05)
		B (EV)	10	-0.09 (-0.12, -0.06)
	Cheng, R., 2022 (36)	A (Amuc_1100)	3	28.23 (20.34, 36.12)
		B (Amuc_1100 Δ80)	3	23.29 (19.49, 27.09)
	Cheng, R., 2021 (37)	B (Amuc_1100)	3	73.98 (69.86, 78.10)
	Sun, Y., 2024 (34)	A (*A. muciniphila,* live)	8	2.08 (-1.57, 5.73)
		B (Amuc_1100)	8	11.80 (6.99, 16.61)
	Ding, Y., 2021 (33)	*A. muciniphila* (live)	6	4.62 (-12.97, 22.21)
	Wang, J., 2021 (42)	B (Amuc_1100)	3	23.08 (19.88, 26.28)
	Wu, F., 2019 (30)	*A. muciniphila* (live)	10	-2.30 (-24.91, 20.31)
**Overall P-value**				16.94 (2.38, 31.49); 0.02
Serum Corticosterone	Cheng, R., 2022 (36)	A (Amuc_1100)	3	-4.76 (-10.08, 0.56)
		B (Amuc_1100 Δ80)	3	-30.95 (-34.31, -27.59)
	Sun, Y., 2024 (34)	A (*A. muciniphila,* live)	8	-174.59 (-216.79, -132.39)
		B (Amuc_1100)	8	-82.70 (-185.38, 19.98)
	Ding, Y., 2021 (33)	*A. muciniphila* (live)	6	-8.69 (-16.40, -0.98)
**Overall P-value**				-56.42 (-120.35, 7.52); 0.08
Gut Serotonin	Guo, H., 2023 (43)	A (*A. muciniphila,* live)	12	-1.16 (-23.95, 21.63)
		B (*A. muciniphila,* live)	12	17.21 (4.32, 30.10)

Test	Author, year	Intervention	n/group	SMD (95% CI); P-value
		C (*A. muciniphila,* live)	15	0.02 (-0.00, 0.05)
	Yaghoubfar, R., 2020 (35)	A (A. muciniphila, live)	10	0.03 (-0.00, 0.06)
		B (EV)	10	0.10 (0.07, 0.13)
	Wang, J., 2021 (42)	A (*A. muciniphila,* live)	3	292.18 (44.72, 539.64)
		B (Amuc_1100)	3	340.87 (82.20, 599.54)
**Overall P-value**				91.50 (-9.92, 192.93); 0.08
**Neurotransmitters related factors**				
HIPP BDNF	Guo, D., 2022 (29)	*A. muciniphila* (live)	5	0.29 (-0.12, 0.70)
	Cheng, R., 2022 (36)	A (Amuc_1100)	3	0.50 (0.39, 0.61)
		B (Amuc_1100 Δ80)	3	0.77 (0.62, 0.92)
	Cheng, R., 2021 (37)	B (Amuc_1100)	3	0.10 (-0.04, 0.24)
	Sun, Y., 2024 (34)	A (*A. muciniphila,* live)	6	0.12 (-0.00, 0.24)
		B (Amuc_1100)	6	0.22 (0.12, 0.32)
	Ding, Y., 2021 (33)	*A. muciniphila* (live)	6	0.46 (0.18, 0.74)
**Overall P-value**				0.35 (0.16, 0.54); <0.001
HIPP CREB1	Cheng, R., 2022 (36)	A (Amuc_1100)	3	0.04 (-0.00, 0.08)
		B (Amuc_1100 Δ80)	3	0.19 (0.07, 0.31)
	Cheng, R., 2021 (37)	B (Amuc_1100)	3	0.36 (0.12, 0.60)
**Overall P-value**				0.17 (-0.00, 0.33); 0.05
HIPP GR	Cheng, R., 2022 (36)	A (Amuc_1100)	3	-0.34 (-0.37, -0.31)
		B (Amuc_1100 Δ80)	3	-0.48, (-0.51, -0.45)
	Sun, Y., 2024 (34)	A (A. muciniphila)	6	-0.49 (-0.81, -0.17)
		B (Amuc_1100)	6	-0.46 (-0.76, -0.16)
**Overall P-value**				-0.42 (-0.52, -0.32); <0.001
Serum DAO	Chen, Y., 2023 (39)		6	-1.45 (-1.84, -1.06)

Test	Author, year	Intervention	n/group	SMD (95% CI); P-value
	Ou, Z., 2020 (41)	A (*A. muciniphila,* live)	8	-29.34 (-49.56, -9.12)
		B (*A. muciniphila,* live)	8	-6.53 (-24.62, 11.56)
**Overall P-value**				-10.16 (-25.92, 5.61); 0.21
Gut TPH1	Guo, H., 2023 (43)	A (*A. muciniphila,* live)	12	0.07 (-0.46, 0.60)
		B (*A. muciniphila*) *,* live	12	-0.11 (-0.49, 0.27)
		C (*A. muciniphila,* live)	15	-0.56 (-1.11, -0.01)
	Yaghoubfar, R., 2020 (35)	A (A. muciniphila, live)	10	0.98 (0.65, 1.31)
		B (EV)	10	3.43 (2.90, 3.96)
	Cheng, R., 2021 ()	37B (Amuc_1100)	3	0.82 (0.55, 1.09)
**Overall P-value**				0.77 (-0.36, 1.90); 0.18
**Neuroinflammation**				
IL-1β	Guo, D., 2022 (29)	*A. muciniphila* (live)	5	-2.40 (-5.76, 0.96)
	Cheng, R., 2022 (36)	A (Amuc_1100)	3	-2.20 (-2.54, -1.86)
		B (Amuc_1100 Δ80)	3	-2.90 (-3.21, -2.59)
	Cheng, R., 2021 (37)	A (Amuc_1100)	3	-5.62 (-7.15, -4.09)
	Yang, Y., 2019 (38)	*A. muciniphila* (live)	8	-1.54 (-2.29, -0.79)
	Chen, Y., 2023 (39)	*A. muciniphila* (live)	6	-1.33 (-1.70, -0.96)
**Overall P-value**				-2.58 (-3.79, -1.36); <0.001
IL-6	Cheng, R., 2022 (36)	A (Amuc_1100)	3	-0.77 (-1.03, -0.51)
		B (Amuc_1100 Δ80)	3	-1.36 (-1.62, -1.10)
	Cheng, R., 2021 (37)	A (Amuc_1100)	3	-1.20 (-1.45, -0.95)
	Yang, Y., 2019 (38)	*A. muciniphila* (live)	8	-1.13 (-1.85, -0.41)
**Overall P-value**				-1.11 (-1.40, -0.82); 0.01
TNFα	Guo, D., 2022 (29)	*A. muciniphila* (live)	5	-8.28 (-13.35, -3.21)
	Cheng, R., 2022 (36)	A (Amuc_1100)	3	-0.47 (-0.79, -0.15)

Test	Author, year	Intervention	n/group	SMD (95% CI); P-value
		B (Amuc_1100 Δ80)	3	-1.12 (-1.30, -0.94)
	Cheng, R., 2021 (37)	A (Amuc_1100)	3	-1.53 (-1.57, -1.49)
	Yang, Y., 2019 (38)	*A. muciniphila* (live)	8	-2.16 (-3.10, -1.22)
	Chen, Y., 2023 (39)	*A. muciniphila* (live)	6	-20.00 (-27.30, -12.70)
**Overall P-value**				-4.87 (-10.12, 0.38); 0.07
HIPP Iba1	Guo, D., 2022 (29)	*A. muciniphila* (live)	5	-2.54 (-4.55, -0.53)
	Li, N., 2023 (40)	*A. muciniphila* (live)	16	-106.67 (-125.14, -88.20)
	Yang, Y., 2019 (38)	*A. muciniphila* (live)	8	-77.89 (-108.34, -47.77)
	Chen, Y., 2023 (39)	*A. muciniphila* (live)	6	-2.17 (-2.64, -1.70)
**Overall P-value**				-46.22 (-98.70, 6.25); 0.08
**Systemic Inflammation**				
IL-1β	Cheng, R., 2022 (36)	A (Amuc_1100)	3	-20.00 (-25.66, -14.34)
		B (Amuc_1100 Δ80)	3	-38.40 (-44.65, -32.15)
**Overall P-value**				-29.15 (-47.18, -11.12); <0.001
IL-6	Guo, D., 2022 (29)	*A. muciniphila* (live)	5	-51.72 (-88.11, -15.33)
	Cheng, R., 2022 (36)	A (Amuc_1100)	3	-11.77 (-15.99, -7.55)
		B (Amuc_1100 Δ80)	3	-19.61 (-28.38, -10.84)
**Overall P-value**				-18.17 (-29.29, -7.06); <0.001
TNFα	Guo, D., 2022 (29)	*A. muciniphila* (live)	5	-36.55 (-88.57, 15.47)
	Cheng, R., 2022 (36)	A (Amuc_1100)	3	-88.23 (-119.91, -56.55)
		B (Amuc_1100 Δ80)	3	-88.23 (-122.54, -53.92)
**Overall P-value**				-78.54 (-102.30, -54.77); <0.001
**Tight junction proteins expression in gut**				
Claudin expression	Guo, D., 2022 (29)	*A. muciniphila* (live)	5	0.15 (-0.19, 0.49)
	Meynier, M., 2024 (31)	A (*A. muciniphila*, pasteurized)	12	-0.73 (-1.27, -0.19)

Test	Author, year	Intervention	n/group	SMD (95% CI); P-value
		B (*A. muciniphila,* pasteurized)	12	-1.06 (-1.58, -0.54)
**Overall P-value**				-0.52 (-1.25, 0.21); 0.16
Ocl expression	Guo, D., 2022 (29)	*A. muciniphila* (live)	5	0.74 (0.11, 1.37)
	Yang, Y., 2019 (34)	*A. muciniphila* (live)	8	0.37 (0.14, 0.60)
	Meynier, M., 2024 (31)	A (*A. muciniphila,* pasteurized)	12	-0.35 (-1.49, 0.79)
		B (*A. muciniphila,* pasteurized)	12	-0.88 (-1.72, -0.04)
		C (*A. muciniphila,* pasteurized)	12	-0.03 (-0.35, 0.29)
		D (*A. muciniphila,* pasteurized)	12	-0.20 (-0.52, 0.12)
**Overall P-value**				0.02 (-0.37, 0.41); 0.93

Sub-group analysis results

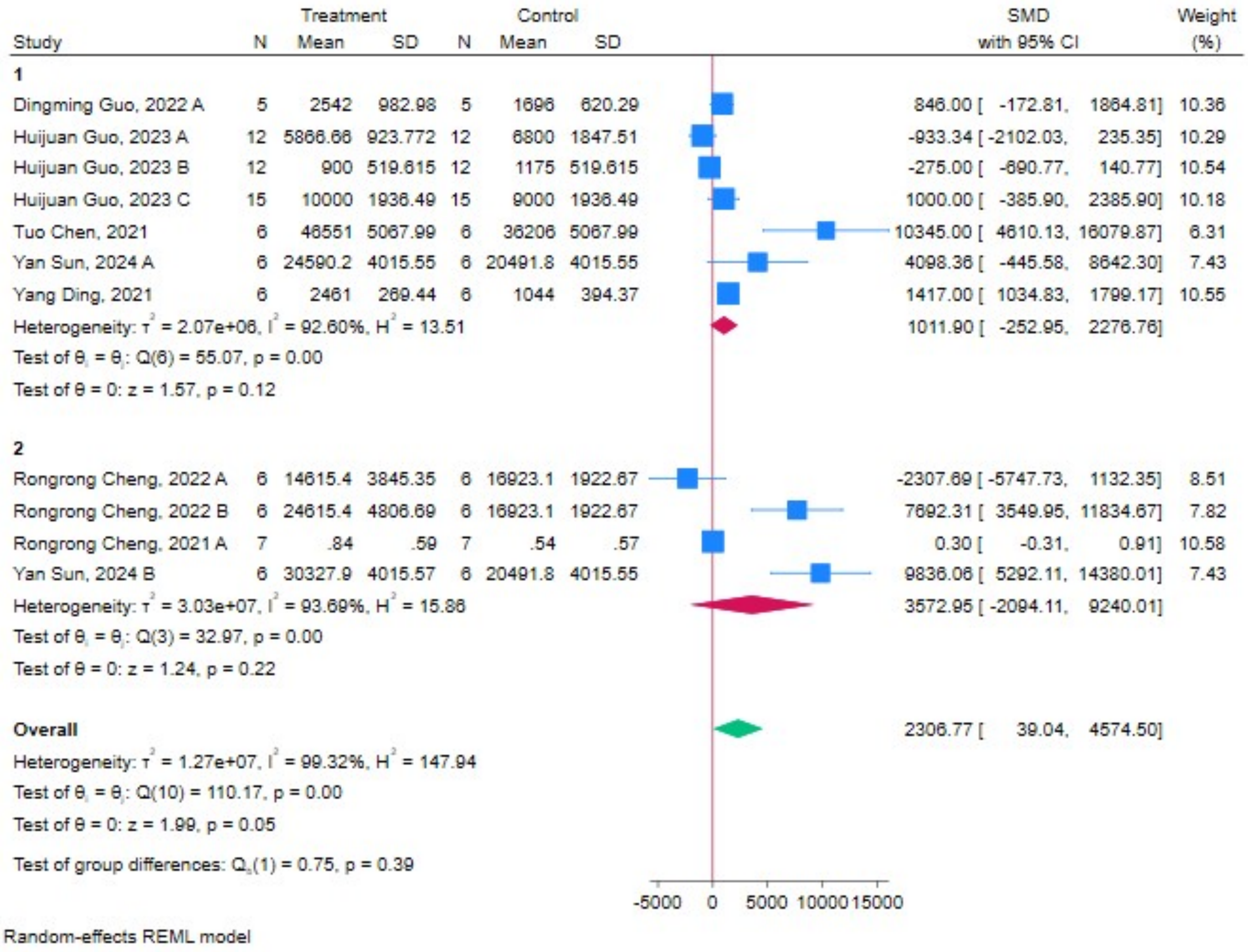

Figure S1. Subgroup analysis for OFT test for live (1) and non-alive *A. muciniphila* (2) interventions.

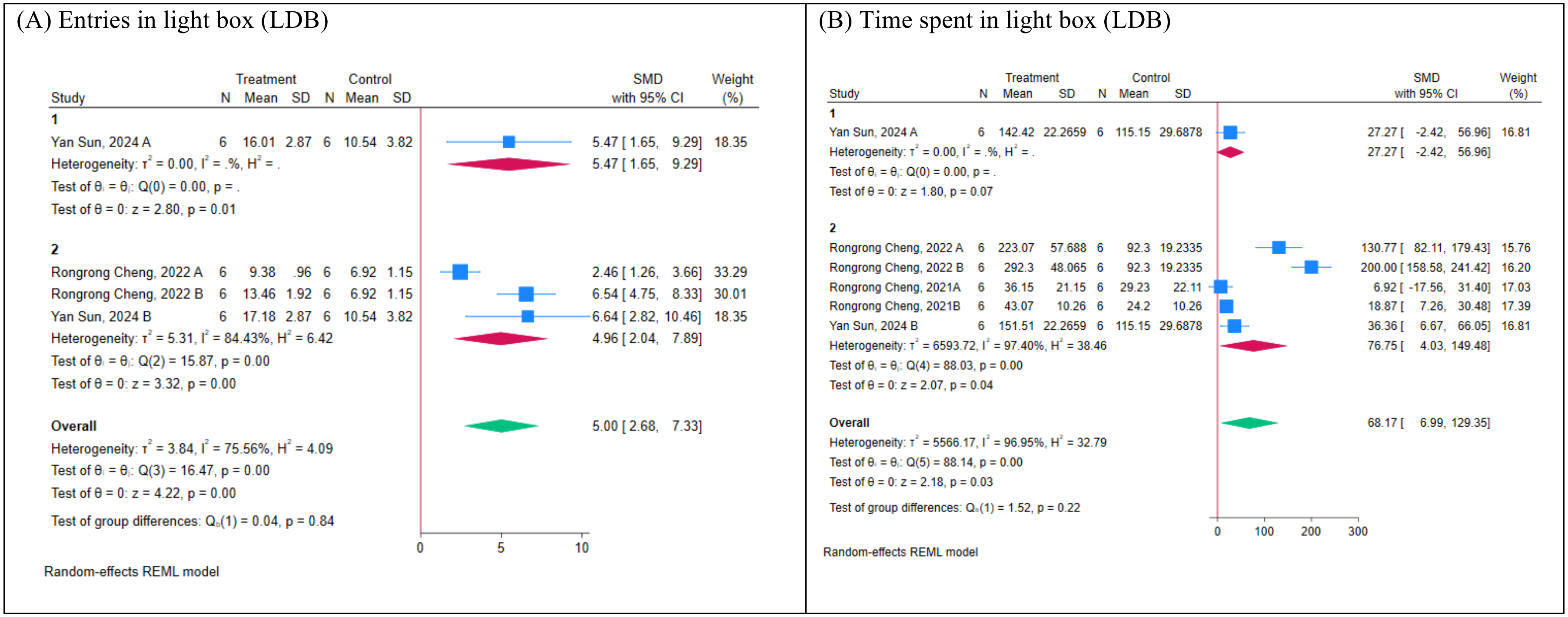

Figure S2. Subgroup analysis for entries (A) and time spent (B) in the lightbox for LDB test for live (1) and non-live *A. muciniphila* (2) interventions.

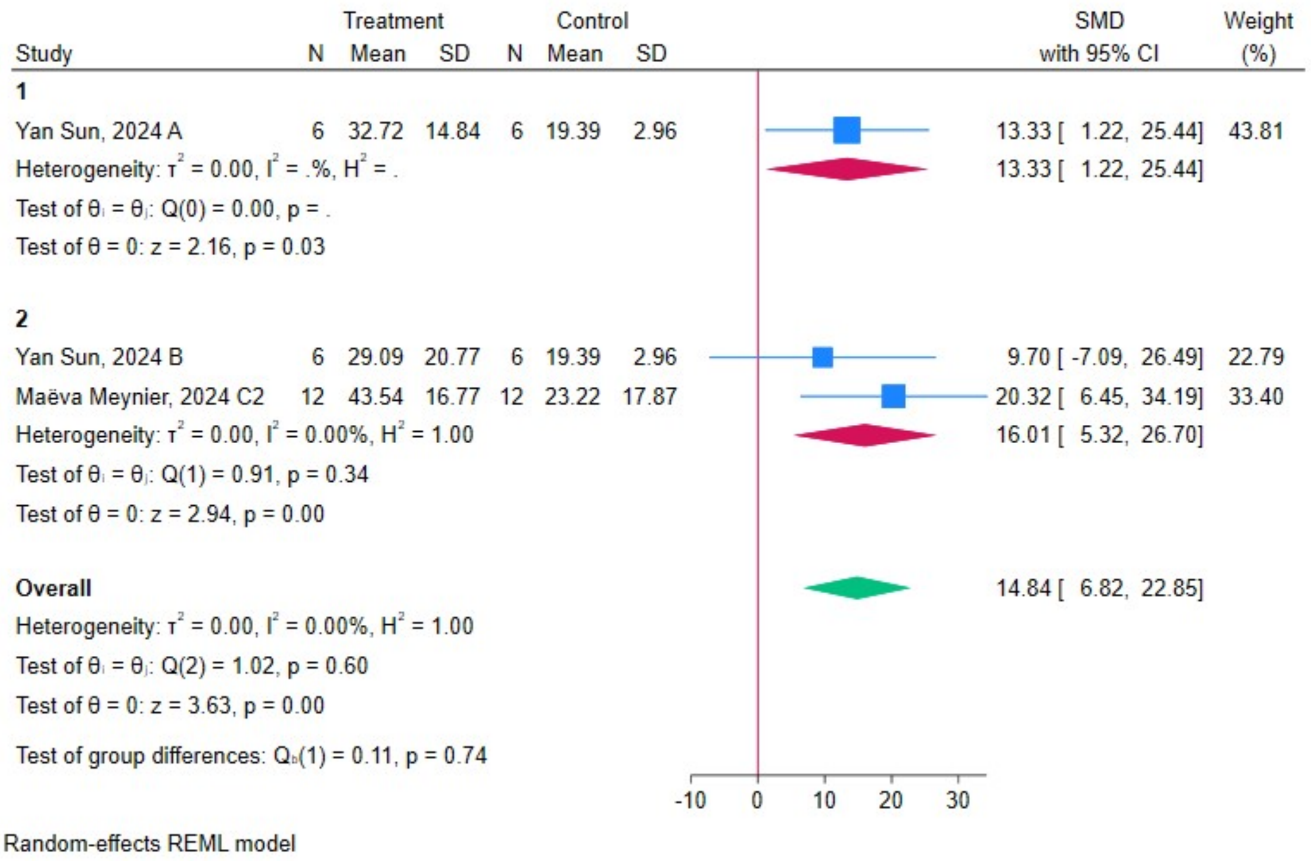

Figure S3. Subgroup analysis for time spent in the open arms of the EPM for live (1) and non-alive *A. muciniphila* (2) interventions.

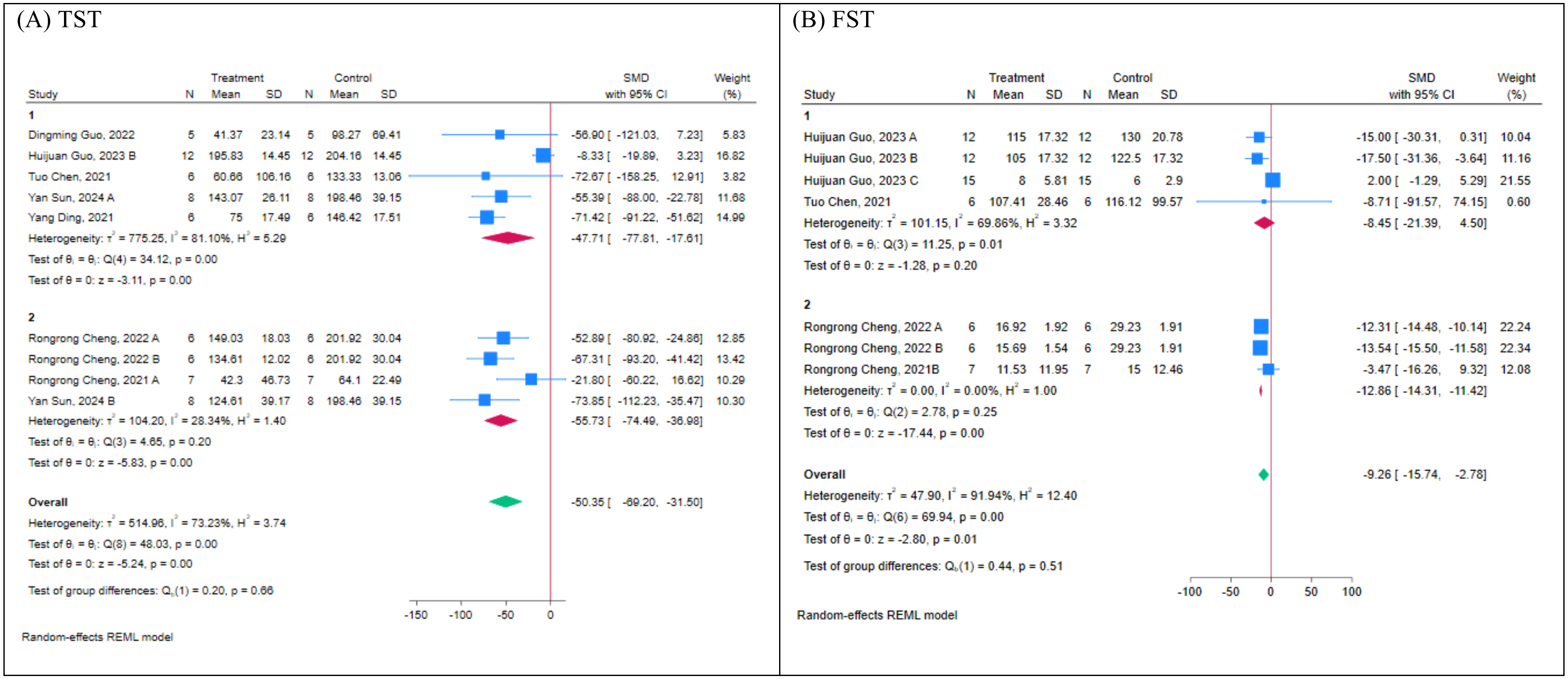

Figure S4. Subgroup analysis for immobility time in TST (A) and FST (B) for live (1) and non-live *A. muciniphila* (2) interventions.

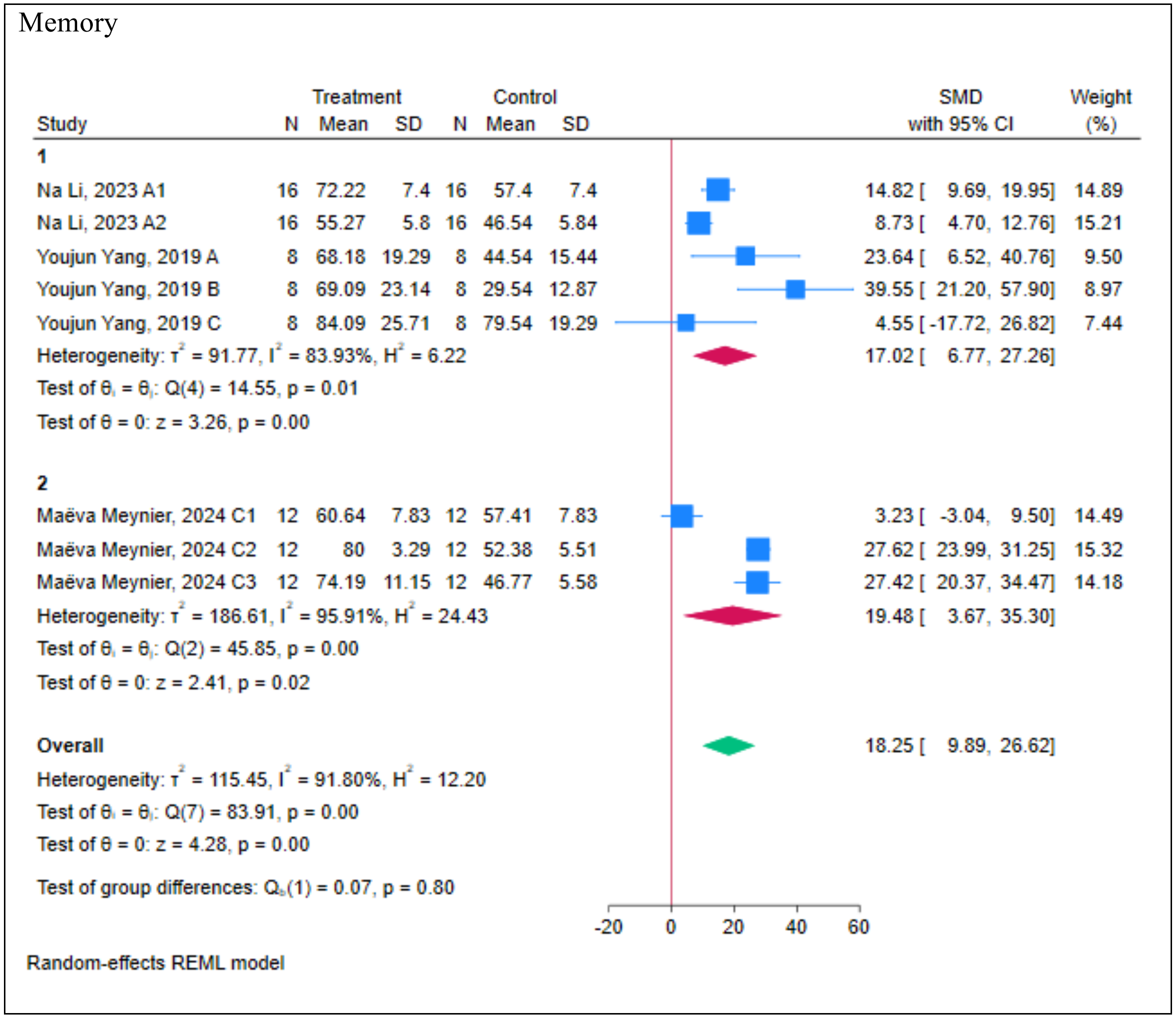

Figure S5. Subgroup analysis for memory tests for live (1) and non-alive *A. muciniphila* (2) interventions.

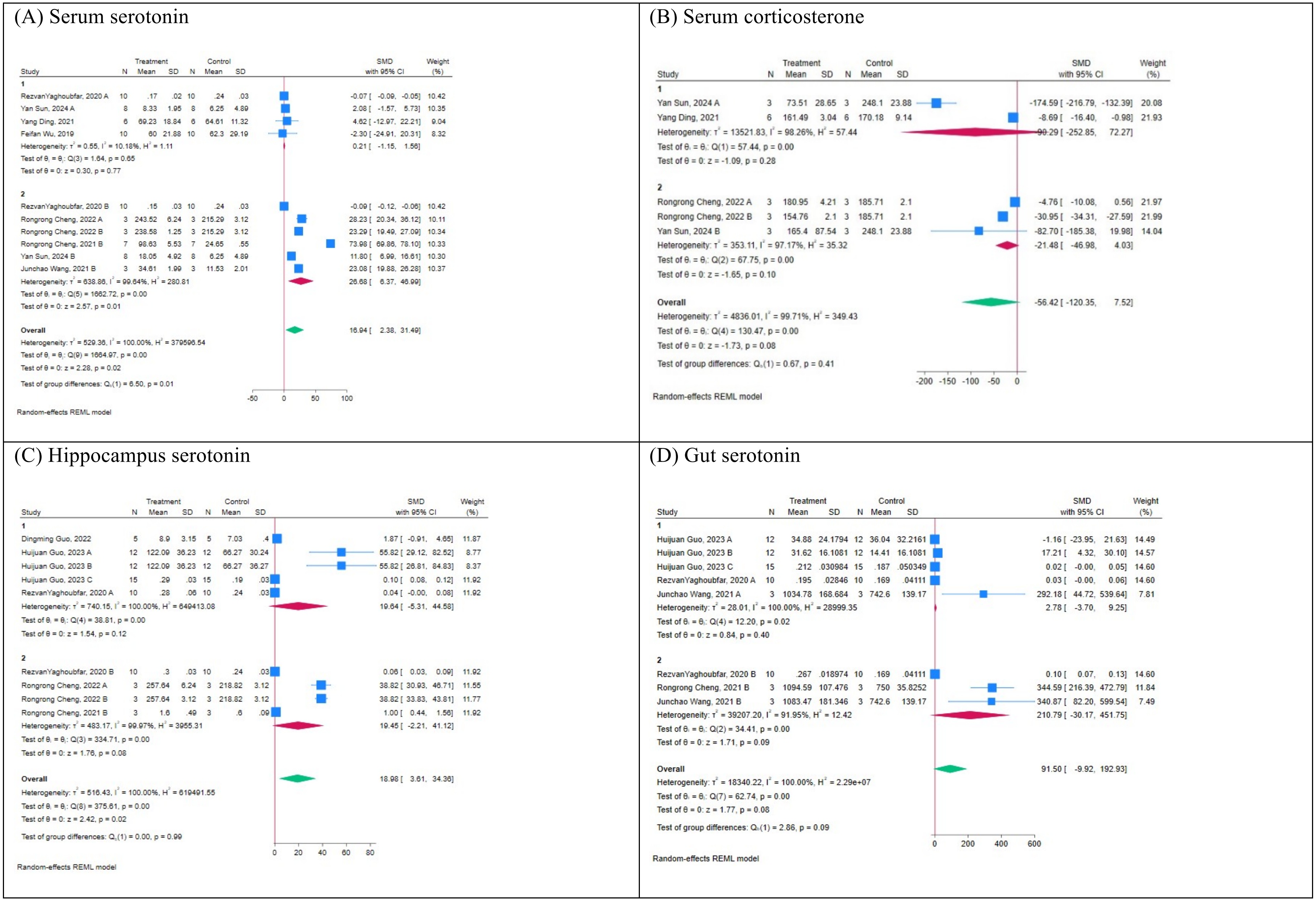

Figure S6. Subgroup analysis of serum serotonin (A) and corticosterone (B), hippocampus serotonin (C) and gut serotonin (D) level for live (1) and non-alive *A. muciniphila* (2) interventions.

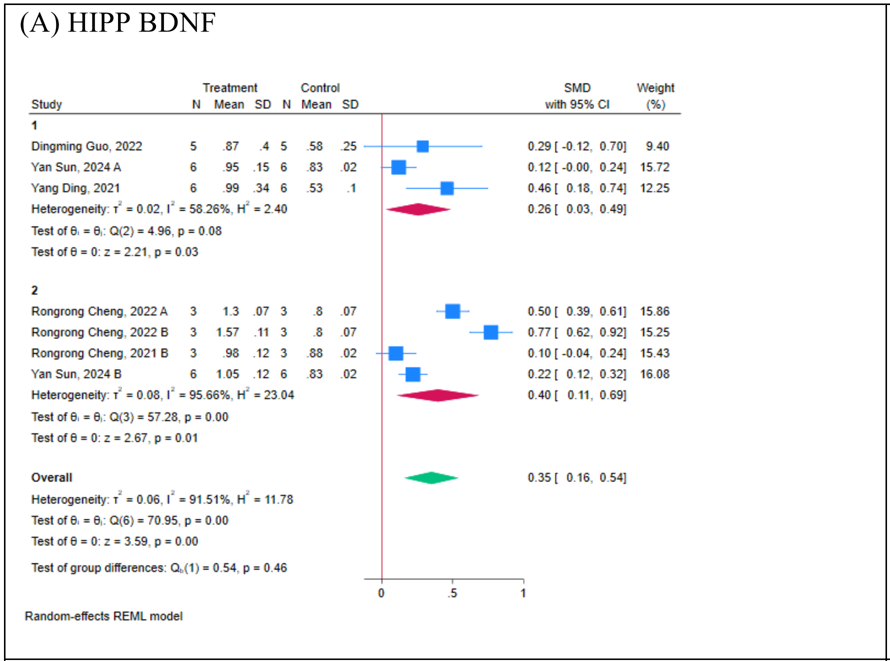

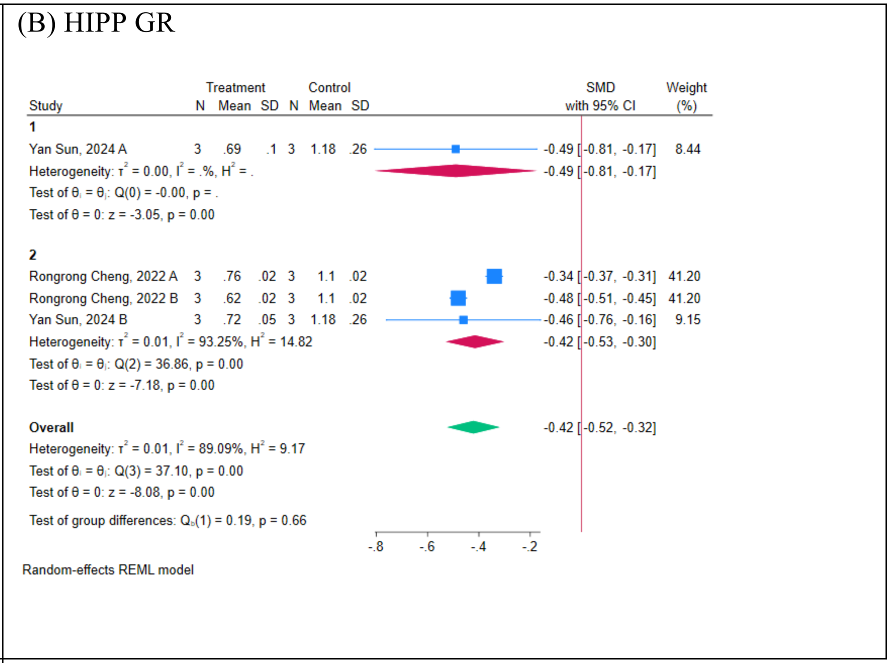

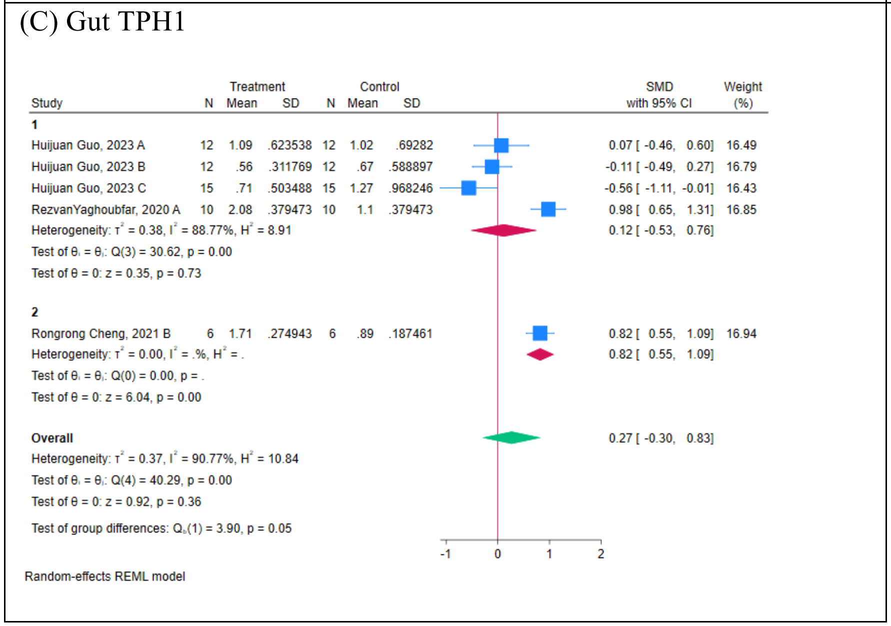

Figure S7. Subgroup analysis of neurotransmitter-related factors. The levels of BDNF (A) and GR (B) in the HIPP and TPH1 in the gut (C) were analyzed for live (1) and non-live (2) *A. muciniphila* interventions.

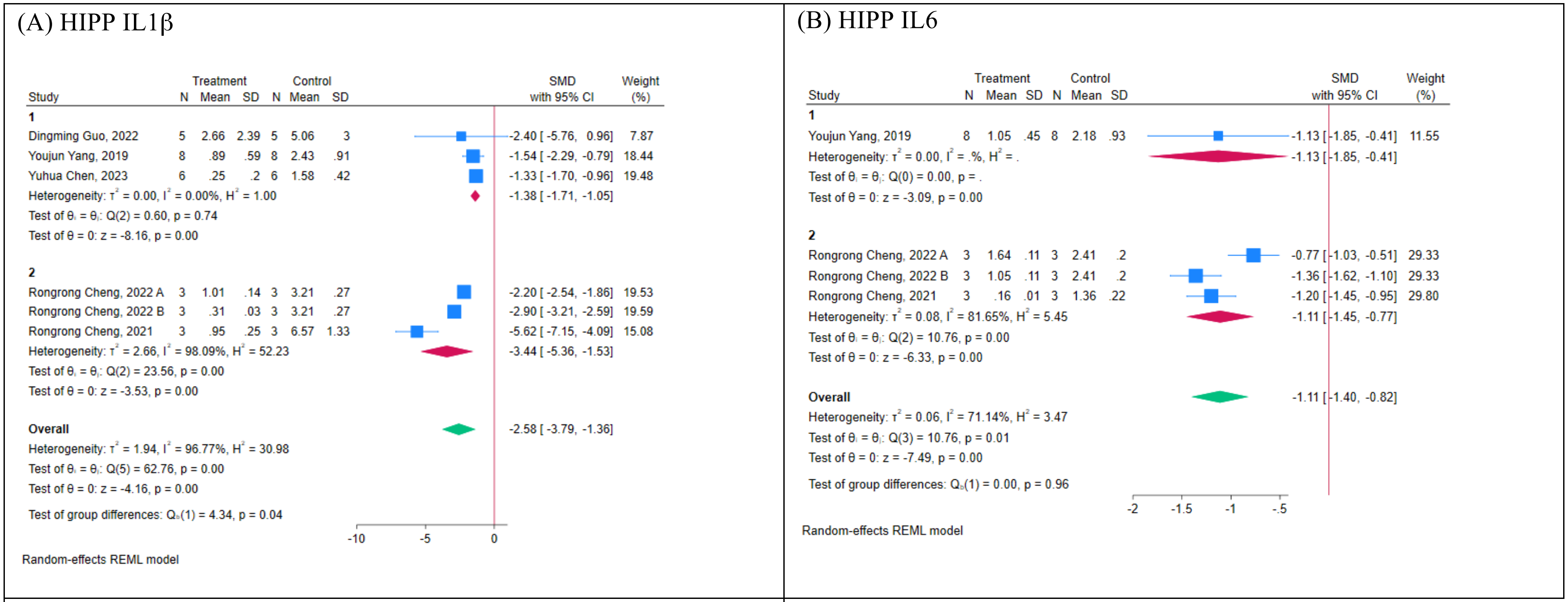

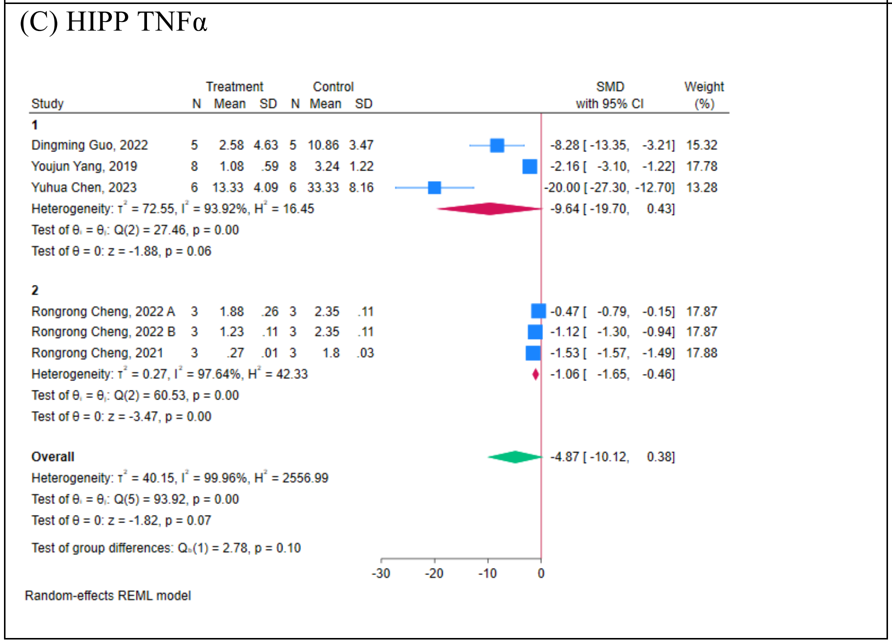

Figure S8. Subgroup analysis of IL1β (A), IL6 (B) and TNFα (C) levels in the hippocampus for live (1) and non-alive *A. muciniphila* (2) interventions.

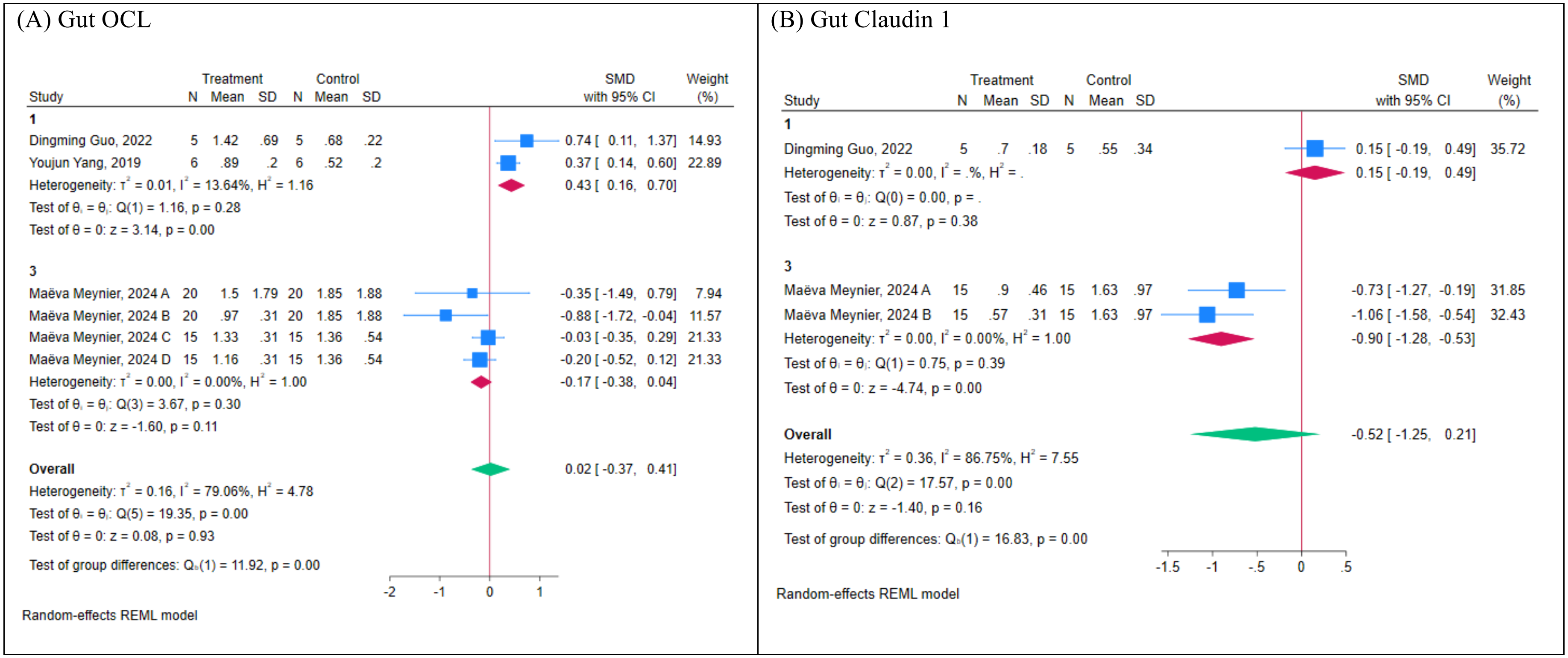

Figure S9. Subgroup analysis of the tight junction proteins Ocl (A) and claudin 1 for live (1) and non-alive *A. muciniphila* (2) interventions.
